# Emergência nutricional no território Yanomami: ações desenvolvidas para enfrentamento da fome e sua interface com os determinantes comerciais da saúde

**DOI:** 10.1590/0102-311XPT073525

**Published:** 2025-12-01

**Authors:** Letícia de Freitas Portugal, Thamillys Rodrigues Souza, Luciene Burlandy, Melissa Mialon, Beatriz Gouveia Moura, Mayara Barroso Quintanilha, Kimielle Cristina Silva, Paulo Cesar de Castro, Inês Rugani Ribeiro de Castro, Camila Maranha Paes de Carvalho

**Affiliations:** 1 Universidade Federal Fluminense, Niterói, Brasil.; 2 Observatório Brasileiro de Conflitos de Interesses em Alimentação e Nutrição, Rio de Janeiro, Brasil.; 3 Université Paris Cité, Paris, France.; 4 Universidade do Estado do Rio de Janeiro, Rio de Janeiro, Brasil.; 5 Ministério da Saúde, Brasília, Brasil.; 6 Universidade Federal do Rio de Janeiro, Rio de Janeiro, Brasil.

**Keywords:** Povos Indígenas, Segurança Alimentar, Fome, Determinantes Comerciais da Saúde, Indigenous Peoples, Food Security, Hunger, Commercial Determinants of Health, Pueblos Indígenas, Seguridad Alimentaria, Hambre, Determinantes Comerciales de la Salud

## Abstract

A Terra Indígena Yanomami, a maior do Brasil, recebeu uma operação emergencial para enfrentar a crise sanitária devido à morte de 570 crianças menores de 5 anos por causas evitáveis entre 2019 e 2022, resultante do garimpo ilegal e desestruturação do sistema de saúde. A partir de janeiro de 2023, foi declarada situação de Emergência em Saúde Pública de Importância Nacional nesse território. O objetivo deste estudo foi analisar as ações de alimentação e nutrição realizadas por diferentes atores nos anos de 2023 e 2024, à luz dos determinantes comerciais da saúde. Por meio de análise documental e de dados secundários, foram mapeados 14 atores comerciais, 27 organizações não governamentais e dois de organismos internacionais. Dentre as 35 ações identificadas, prevaleceram a distribuição de alimentos (n = 16, 45,7%) e campanhas para arrecadar recursos financeiros (n = 8, 22,8%), sendo que a maioria (n = 27, 77,1%) ocorreu por meio de interações entre os atores. Em 81,3% (n = 13) das doações de alimentos, não foram descritos os gêneros alimentícios, mas houve doação de alimentos ultraprocessados. Os atores comerciais buscaram se posicionar como “parceiros” no momento de crise humanitária, agindo em conjunto com atores não governamentais que atuam em situações emergenciais. Entretanto, houve falta de transparência na maioria das iniciativas, sem a descrição dos alimentos doados ou do uso dos valores doados. Os resultados indicam fatores associados com ações de gestão da reputação e *socialwashing* realizadas pelos atores comerciais e pelas organizações não governamentais.

## Abstract (en)

The Yanomami Indigenous Territory is the largest in Brazil and was the stage of an emergency operation to address a health crisis involving the deaths of 570 children under five years of age from preventable causes between 2019 and 2022, resulting from illegal mining and the dismantling of the health system. In January 2023, a Public Health Emergency of National Concern was declared in this territory. The objective of the study was to analyze food and nutrition initiatives carried out by different actors in 2023 and 2024 in light of the commercial determinants of health. Through the analysis of documents and secondary data, 14 commercial actors, 27 nongovernmental organizations, and two international organizations were mapped. Among the 35 actions identified, food distribution (n = 16, 45.7%) and fundraising campaigns (n = 8, 22.8%) predominated and the majority (n = 27, 77.1%) occurred through interactions among the actors. In 81.3% (n = 13) of the food donations, the types of food were not described, but the donation of ultra-processed foods occurred. Commercial actors sought to position themselves as “partners” during the humanitarian crisis, working alongside nongovernmental actors who responded to the emergency situation. However, most initiatives lacked transparency, with no description of the foods donated or how the funds were used. The results indicate factors associated with reputation management and social washing practices carried out by commercial actors and nongovernmental organizations.

## Abstract (es)

La Tierra Indígena Yanomami es la mayor de Brasil y recibió una operación de emergencia para enfrentar la crisis sanitaria debido a la muerte de 570 niños menores de 5 años por causas evitables entre 2019 y 2022 derivadas de la minería ilegal y la desestructuración del sistema de salud. La operación se llevó a cabo a partir de enero de 2023, con la declaración de la situación de Emergencia en Salud Pública de Importancia Nacional en este territorio. El objetivo del estudio fue analizar las acciones de alimentación y nutrición realizadas por diferentes actores en los años 2023 y 2024 a la luz de los determinantes comerciales de la salud. Por medio de análisis documental y de datos secundarios, se mapearon 14 actores comerciales, 27 organizaciones no gubernamentales y dos de organismos internacionales. Entre las 35 acciones identificadas, prevalecieron la distribución de alimentos (n = 16, 45,7%) y las campañas para recaudar recursos financieros (n = 8, 22,8%) y la mayoría (n = 27, 77,1%) ocurrió por medio de interacciones entre los actores. En el 81,3% (n = 13) de las donaciones de alimentos, no se describieron los géneros alimenticios, pero hubo donación de alimentos ultraprocesados. Los actores comerciales buscaron posicionarse como “aliados” en el momento de crisis humanitaria, actuando en conjunto con actores no gubernamentales que actúan en situaciones de emergencia. Sin embargo, faltó transparencia en la mayoría de las iniciativas, sin la descripción de los alimentos donados o de cómo fueron utilizados los valores donados. Los resultados indican factores asociados con acciones de gestión de la reputación y *socialwashing* realizadas por los actores comerciales y por las organizaciones no gubernamentales.

## Introdução

A Declaração das Nações Unidas sobre os Direitos dos Povos Indígenas afirma seu direito pleno à saúde e seguridade social e os reconhecem como participantes ativos da elaboração de estratégias sobre suas práticas tradicionais ^1^. Apesar disso, esses povos constituem um dos segmentos mais vulnerabilizados da população. Vivem em contextos de desigualdades sociais e de saúde, resultantes de processos de colonização, desapropriação, marginalização e ruptura de culturas ^2^ que provocam e/ou ampliam violações de direitos humanos ^3^.

A interação entre diferentes determinantes sociais, ambientais, culturais, econômicos e raciais ^4^ pode impactar positiva ou negativamente a saúde indígena ^5^. Os atores comerciais, como empresas, corporações e demais agentes do setor privado que promovem os interesses das indústrias, influenciam a determinação social do processo saúde-doença ^6^, e o conceito de Determinantes Comerciais da Saúde ^7^ refere-se aos meios, sistemas e práticas que esses atores utilizam para interferir em políticas e às ações públicas que podem afetar a promoção da saúde e da equidade.

Globalmente, estudos sobre determinantes comerciais da saúde e saúde indígena são incipientes ^8^ ^,^ ^9^, mas apontam duas problemáticas relacionadas com atores comerciais e alimentação e nutrição: a perda da alimentação tradicional devido à atuação da indústria de alimentos ultraprocessados e a perda dos saberes tradicionais decorrente da atuação da indústria de fórmulas infantis ^8^ ^,^ ^10^.

No Brasil, a violação de direitos indígenas ocorre desde a colonização, agravada pela neocolonização e pela expansão do neoextrativismo oriundo de garimpeiros, madeireiros, dentre outros ^3^. Há uma dificuldade de conhecer suas condições de saúde e uma necessidade de ampliar as informações sobre seus determinantes para visibilizar suas diferentes realidades ^5^. A situação alimentar e nutricional dessas populações, decorrente da falta de acesso a uma alimentação adequada e saudável, amplia sua vulnerabilidade em saúde e reflete suas condições de vida ^11^.

A Terra Indígena (TI) Yanomami é a maior do país e possui cerca de 31 mil habitantes ^12^ que, em 2021, já apresentavam casos de desnutrição grave ^3^ ^,^ ^13^. Em janeiro de 2023, o Ministério da Saúde declarou a Emergência em Saúde Pública de Importância Nacional (ESPIN) após a divulgação da morte de 570 crianças de até cinco anos por causas evitáveis, constituindo crise humanitária ^12^ ^,^ ^14^ ^,^ ^15^. O Governo Federal implementou um grupo de trabalho para responder à situação e lidar com demandas emergenciais e problemas crônicos de infraestrutura, logística e recursos humanos ^14^. Paralelamente, diferentes setores procuraram se engajar nesse processo ^16^ ^,^ ^17^ ^,^ ^18^.

O governo brasileiro publicou um documento que padronizou e especificou alimentos e insumos a serem distribuídos na TI Yanomami com uma listagem de itens não perecíveis, como peixe em conserva (sardinha), e perecíveis, como macaxeira e batata-doce, que fazem parte da cultura alimentar desses povos ^19^. A finalidade foi orientar as ações de combate à situação de ESPIN, em decorrência de desassistência aos indígenas ^19^.

As análises de soluções apresentadas às complexas e diversas violações do direito humano à alimentação adequada (DHAA) dos povos indígenas, na perspectiva dos determinantes comerciais da saúde, são incipientes, e não foram identificadas pesquisas deste tipo no Brasil. Portanto, este estudo investigou e analisou as respostas propostas por atores comerciais, organizações não governamentais (ONGs) e organismos internacionais, no âmbito da alimentação e nutrição, diante da situação emergencial vivenciada pelos Yanomami em 2023, em sua interface com a segurança alimentar e nutricional e o DHAA na perspectiva dos determinantes comerciais da saúde. A pergunta que orientou a pesquisa foi: como essas respostas potencialmente se relacionam com os determinantes comerciais da saúde e com os objetivos que orientam a Política Nacional de Alimentação e Nutrição (PNAN) e a Política Nacional de Segurança Alimentar e Nutricional (PNSAN)?

## Métodos

### Matriz conceitual

À luz da abordagem dos determinantes comerciais da saúde ^7^, foram analisadas as respostas, no âmbito da alimentação e nutrição, dos atores comerciais, de ONGs e organismos internacionais à emergência Yanomami em 2023, bem como as interações entre esses atores.

Foram destacadas as práticas de gestão da reputação dos atores comerciais, concebidas como esforços para moldar a legitimidade e a credibilidade, e aprimorar a imagem da marca corporativa ^7^, agrupadas em duas categorias principais: (a) responsabilidade social corporativa e ESG (do inglês *environmental, social, governance*) − conceitos semelhantes que envolvem atores comerciais que assumem compromissos voluntários para defender as normas éticas, abster-se de regulações governamentais e de causar danos à saúde humana e ao planeta; e (b) institucionalização de parcerias público-privadas, nas quais atores governamentais e atores comerciais são envolvidos em redes de governança com mecanismos fracos de aplicação e falta de controle democrático. As práticas dos diferentes tipos de atores influenciam a saúde e a equidade em saúde de modos distintos ^7^. O estudo analisou como essas práticas potencialmente interferiram nesses dois aspectos.

Foram problematizados os tipos de alimentos oferecidos, com base em princípios, objetivos, diretrizes e concepções que orientam a PNAN, a PNSAN e o *Guia Alimentar para a População Brasileira*, com destaque para os conceitos de segurança alimentar e nutricional ^20^, soberania alimentar ^21^, alimentação adequada e saudável ^10^ e DHAA ^22^. A nota técnica que especificou os alimentos prioritários para serem distribuídos na TI Yanomami ^19^ também foi considerada.

A análise, fundamentada nessa matriz conceitual (Quadro 1), aprofundou a compreensão sobre saúde e equidade no âmbito dos sistemas alimentares agroindustriais, articulada ao conceito de sindemia global de desnutrição, obesidade e mudanças climáticas ^23^. Desenvolveu-se uma análise integrada da alimentação, nutrição e dos determinantes comerciais da saúde, a partir de uma perspectiva sistêmica ^23^, pressupondo que as respostas a situações emergenciais formuladas nessa perspectiva consideram as relações entre os componentes da sindemia, além das desigualdades e violações à soberania alimentar dos povos. Portanto, o estudo analisou como as diferentes respostas repercutiram nos distintos fatores sinérgicos.

**Quadro 1:** Matriz dos conceitos utilizados para a fundamentação da análise do estudo.

CONCEITO	DEFINIÇÃO	DOCUMENTO ORIGINAL
Segurança alimentar e nutricional	“*Realização do direito de todos ao acesso regular e permanente a alimentos de qualidade, em quantidade suficiente, sem comprometer o acesso a outras necessidades essenciais, tendo como base práticas alimentares promotoras de saúde que respeitem a diversidade cultural e que sejam ambiental, cultural, econômica e socialmente sustentáveis*”	Lei Orgânica de Segurança Alimentar e Nutricional ^20^
Soberania alimentar	“*A soberania alimentar é um direito dos povos a alimentos nutritivos e culturalmente adequados, acessíveis, produzidos de forma sustentável e ecológica, e seu direito de decidir seu próprio sistema alimentício e produtivo. Isto coloca aqueles que produzem, distribuem e consomem alimentos no coração dos sistemas e políticas alimentares, por cima das exigências dos mercados e das empresas*”	Declaração de Nyéléni ^21^ (p. 1)
Alimentação adequada e saudável	“*Um direito humano básico que envolve a garantia ao acesso permanente e regular, de forma socialmente justa, a uma prática alimentar adequada aos aspectos biológicos e sociais do indivíduo e que deve estar em acordo com as necessidades alimentares especiais; ser referenciada pela cultura alimentar e pelas dimensões de gênero, raça e etnia; acessível do ponto de vista físico e financeiro; harmônica em quantidade e qualidade, atendendo aos princípios da variedade, equilíbrio, moderação e prazer; e baseada em práticas produtivas adequadas e sustentáveis*”	Guia Alimentar para a População Brasileira ^10^ (p. 8)
Direito humano à alimentação adequada e saudável	“*Deve reconhecer a diversidade dos povos indígenas e dos povos e comunidades tradicionais, tendo como base o Decreto nº 6.040/2007, com vistas a promover e respeitar a matriz produtora de alimentos destes grupos e com respeito à sua diversidade cultural e alimentar*”	Manifesto da 5ª Conferência Nacional de Segurança Alimentar e Nutricional à Sociedade Brasileira sobre Comida de Verdade no Campo e na Cidade, por Direitos e Soberania Alimentar ^22^ (p. 2)

Fonte: elaboração própria.

Grupos de baixa renda, indígenas e famílias chefiadas por mulheres são os mais afetados pelos impactos negativos dos sistemas alimentares em suas condições de vida e saúde, incluindo a má nutrição ^24^. Assim, é fundamental analisar as respostas em curto prazo para situações emergenciais, considerando seus efeitos em processos e fatores de médio prazo que contribuem para os problemas sociais, ambientais e de saúde associados aos sistemas alimentares globalizados.

### Métodos de construção e análise de dados

O estudo pautou-se em método de análise documental e análise de dados secundários. Foi realizado um levantamento de documentos de atores envolvidos com a situação emergencial dos Yanomami e o conjunto de documentos analisados foi definido a partir de duas etapas, descritas a seguir.

### Seleção dos atores comerciais

Foi adaptada a metodologia do International Network for Food and Obesity/NCD Research, Monitoring and Action Support (INFORMAS) ^25^ para a seleção inicial dos atores comerciais, previamente à análise documental, com base em dois critérios ^25^: a atuação dos mesmos no Brasil e maior participação de mercado, ambos avaliados por sua posição no ranking da revista *Forbes* no ano de 2023, pela classificação *The Global 2000* ^26^. Foram selecionados 14 atores comerciais do setor de alimentos e de bebidas: os nove primeiros envolvidos na produção de alimentos ultraprocessados ou bebidas açucaradas; três do agronegócio; um do varejo de alimentos e uma rede de *fast-food*.

### Mapeamento dos demais atores

Os demais atores foram incluídos a partir da menção em relatórios ou reportagens analisadas, referentes a ações com relação direta com alimentação e nutrição realizadas pelos atores comerciais previamente selecionados. Em seguida, outros atores foram identificados por meio de um mapeamento baseado na técnica de bola de neve ^27^, a partir da análise dos textos, imagens e vídeos relacionados às ações realizadas quanto à presença de logos, citações diretas, bem como a divulgação de ações realizadas em parceria, pois identificou-se que atores comerciais formaram diferentes coalizões com os demais atores. Desta forma, foram analisados 14 atores comerciais, 27 ONGs e dois organismos internacionais (Material Suplementar − Quadro S1: https://cadernos.ensp.fiocruz.br/static//arquivo/suppl-e00073525_5709.pdf).

### Seleção das fontes de informação

Foram utilizados relatórios anuais, de ESG e de prestação de contas, e publicações dos sites institucionais dos atores comerciais, ONGs e organismos internacionais para análise dos dados de 2023 e 2024 e os seguintes termos de busca em português e seus respectivos na língua inglesa: “Yanomami”; “Fome”; “Indígena” e “Brasil”. Por serem dados institucionais, refletem as práticas, intenções e valores dos atores em questão.

Foram analisadas as publicações (fotos, vídeos e textos) dos perfis institucionais no Instagram dos atores selecionados para o estudo que realizaram ações no âmbito da alimentação e nutrição durante a emergência indígena Yanomami. Essa análise foi incluída pois os diferentes atores costumam usar estes perfis para divulgar suas ações para consumidores e população em geral. A maior acessibilidade de fontes e a crescente midiatização social contribuem para a ampliação da influência, do alcance social e da capacidade performativa das informações inseridas nesses canais ^28^. Assim, foram selecionadas as publicações que descreviam a doação de alimentos ou equipamentos que envolvessem o acesso à água, campanhas para arrecadação de recursos financeiros e/ou alimentos e, divulgação de parcerias para incidir no território Yanomami durante a ESPIN.

### Organização e análise de dados

Todos os dados coletados foram lidos e armazenados em documentos no formato de planilhas, com a ferramenta Google Sheets (https://www.google.com/sheets/about/). Os documentos, incluindo os relatórios e informações coletadas no Instagram, foram salvos em forma de PDF e armazenados na plataforma Google Drive (https://www.google.com/intl/pt-PT/drive/), gerando um backup das ações monitoradas. O roteiro analítico incluiu: nome do documento, objetivos, dados sobre saúde e nutrição e informações da atuação dos atores. A análise dos dados baseou-se em processos de codificação indutivo e dedutivo (orientado pela matriz conceitual e pelos referenciais teóricos do estudo).

Foram sistematizadas, por meio de um esquema gráfico, as interações encontradas no estudo. Os atores monitorados foram representados por suas respectivas logomarcas e, as interações foram sinalizadas por meio de setas.

O foco da coleta foi dados referentes à atuação dos diferentes atores na ESPIN em ações desenvolvidas no âmbito da alimentação e nutrição. Essa etapa foi conduzida entre janeiro e fevereiro de 2025 de forma independente por quatro pesquisadoras (L.F.P., T.R.S., M.B.Q. e B.G.M.).

## Resultados

**Figura 1:**
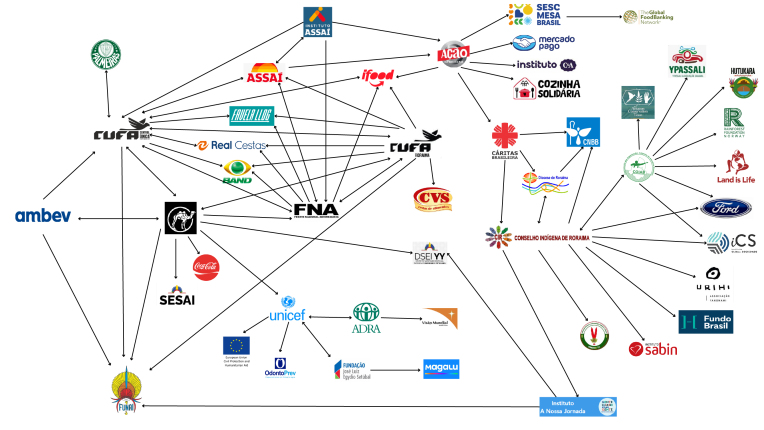
Interações entre atores mapeados no presente estudo que realizaram alguma ação na emergência em saúde pública do povo Yanomami no âmbito da alimentação e nutrição (2023-2024).

Foram analisados 19 relatórios anuais, 37 sites institucionais e 46 perfis institucionais na rede social Instagram. Foram identificados 14 atores comerciais, 27 ONGs e dois de organismos internacionais que se envolveram em ações direcionadas para a emergência indígena Yanomami (Quadro 2 e Figura 1). Além disso, os dados e links de acesso para todas as publicações monitoradas estão disponíveis no Material Suplementar (Quadros S2 e S3: https://cadernos.ensp.fiocruz.br/static//arquivo/suppl-e00073525_5709.pdf).

**Quadro 2:** Caracterização, interações com outros atores e fontes de dados dos atores incluídos no estudo que realizaram alguma ação na emergência em saúde pública do povo Yanomami no âmbito da alimentação e nutrição (2023-2024).

ATOR	ATORES COM QUEM FOI IDENTIFICADO ALGUM TIPO DE INTERAÇÃO	FONTE DE DADOS	LINKS UTILIZADOS
ATORES COMERCIAIS			
Água Camelo	Ambev + CUFA + CUFA Roraima + FNA + FUNAI + SESAI + DSEI Yanomami + Coca-Cola +UNICEF Brasil	Site e Instagram institucional	https://aguacamelo.com.br/ https://www.instagram.com/agua_camelo/
Ambev	Água Camelo + CUFA + FUNAI	Site e Instagram institucional, relatório anual	https://www.ambev.com.br/ https://ri.ambev.com.br/ https://www.instagram.com/aguaama?utm_source=ig_web_button_share_sheet&igsh=ZDNlZDc0MzIxNw==
Assaí Atacadista	CUFA + FNA + Ação da Cidadania + Instituto Assaí	Relatório anual e Instagram institucional	https://ri.assai.com.br/ https://www.instagram.com/assaiatacadistaoficial
Band TV	CUFA + CUFA Roraima + FNA	Site e Instagram institucional	https://www.band.uol.com.br/ https://www.instagram.com/bandtv/
Coca-Cola	Água Camelo	Site e Instagram institucional	https://www.coca-cola.com/br/pt https://www.instagram.com/cocacola_br/ https://www.instagram.com/cocacolafemsa_br/
CVS cestas básicas	CUFA Roraima	Site e Instagram institucional	https://lojacvscesta.com.br/ https://www.instagram.com/cvscestas/
FavelaLLog	CUFA + CUFA Roraima + FNA	Site e Instagram institucional	https://favelallog.com.br/ https://www.instagram.com/favelallog/
Ford	Conselho Indígena de Roraima (CIR) + COIAB	Site e Instagram institucional	https://www.ford.com.br/ https://www.instagram.com/ford/
iFood	CUFA + CUFA Roraima + Ação da Cidadania + FNA	Relatórios de prestação de contas, site e Instagram institucional	https://institucional.ifood.com.br/ https://www.instagram.com/ifoodbrasil/ https://www.instagram.com/ifoodnewsbrasil/ https://www.instagram.com/ifooduniverso/
Magazine Luiza	Fundação José Luiz Egydio Setúbal	Relatório anual, site e Instagram institucional	https://ri.magazineluiza.com.br/show.aspx?idCanal=urUqu4hANldyCLgMRgOsTw== https://www.instagram.com/magazineluiza/
Mercado Pago	Ação da Cidadania	Site e Instagram institucional	https://conexao.mercadopago.com.br/ https://www.instagram.com/mercadopagobr/
OdontoPrev	UNICEF Brasil	Relatório anual, site e Instagram institucional	https://www.odontoprev.com.br/ https://www.instagram.com/odontoprevoficial/
Sociedade Esportiva Palmeiras	CUFA	Site e Instagram institucional	https://www.palmeiras.com.br/ https://www.instagram.com/palmeiras/
Real Cestas	CUFA + CUFA Roraima	Site e Instagram institucional	https://www.realcestas.com.br/ https://www.instagram.com/realcestasoficial/
ATORES DE ORGANIZAÇÕES NACIONAIS E INTERNACIONAIS NÃO GOVERNAMENTAIS			
Ação da Cidadania	iFood + Assaí Atacadista + Instituto Assaí + Cáritas Brasileira + Cozinha Solidária do MTST + Instituto C&A + Mercado Pago + Sesc Mesa Brasil	Relatório anual, site e Instagram institucional	https://www.acaodacidadania.org.br/ https://www.instagram.com/acaodacidadania/
ADRA	UNICEF Brasil + Visão Mundial	Site e Instagram institucional	https://adra.org.br/ https://www.instagram.com/adrabrasil/
ACT-Brasil	COIAB	Site e Instagram institucional	https://www.amazonteam.org/brazil/ https://www.instagram.com/amazonconservationteam/
Associação Hutukara Yanomami	COIAB	Site e Instagram institucional	https://hutukarayanomami.org/ https://www.instagram.com/hutukara_yanomami/
Cáritas Brasileira	Ação da cidadania + CIR + Cepast/CNBB + Diocese de Roraima	Site e Instagram institucional	https://caritas.org.br/ https://www.instagram.com/caritasbrasileira/
Cepast/CNBB	Cáritas Brasileira + Diocese de Roraima + CIR	Site e Instagram institucional	https://cepastcnbb.org.br/ https://www.instagram.com/cepastcnbb/
CIR	Cepast/CNBB + Diocese de Roraima + COIAB + Fundo Brasil + Ford + Instituto Sabin + Instituto A Nossa Jornada + CONDISI-YY + URIHI Associação Yanomami + iCS	Site e Instagram institucional	https://www.cir.org.br/ https://www.instagram.com/cir.oficial/
COIAB	CIR + iCS + Ford + ACT-Brasil + Rainforest Noruega + Land is Life + Associação Hutukara Yanomami + Ypassali Associação Sanuma	Site e Instagram institucional	https://coiab.org.br/ https://www.instagram.com/coiabamazonia/
Cozinha Solidária do MTST	Ação da cidadania	Site e Instagram institucional	https://mtst.org/ https://www.instagram.com/cozinhassolidariasmtst/ https://www.instagram.com/mtstbrasil/
CUFA	Ambev + Assaí Atacadista + Instituto Assaí + Água Camelo + CUFA Roraima + FNA + FavelaLLog + iFood + Band + Palmeiras + Real Cestas + Funai	Site e Instagram institucional	https://cufa.org.br/ https://www.instagram.com/cufabrasil/
CUFA Roraima	Água Camelo + CUFA + FNA + iFood + Assaí atacadista + FavelaLLog + Band + Real Cestas + CVS Cestas Básicas + FUNAI	Instagram institucional	https://www.instagram.com/cufa_rr/
Diocese de Roraima	CIR + Cáritas Brasileira + Cepast/CNBB	Site e Instagram institucional	https://diocesederoraima.org.br/ https://www.instagram.com/diocesederoraima/
FNA	Água Camelo + CUFA + CUFA Roraima + Assaí Atacadista + Instituto Assaí + FavelaLLog + iFood + Band	Instagram institucional	https://www.instagram.com/frentenacionalantirracista/
Fundação José Luiz Egydio Setúbal − Instituto Pensi	UNICEF Brasil + Magazine Luiza	Site e Instagram institucional	https://fundacaojles.org.br/ https://institutopensi.org.br/ https://www.instagram.com/institutopensi/
Fundo Brasil	CIR	Site e Instagram institucional	https://www.fundobrasil.org.br/ https://www.instagram.com/fundobrasil/
Global FoodBanking Network	Sesc Mesa Brasil	Site e Instagram institucional	https://www.foodbanking.org/ https://www.instagram.com/foodbanking/
Instituto A Nossa Jornada	CIR + DSEI Yanomami + Funai	Instagram institucional	https://www.instagram.com/institutoanossajornada?utm_source=ig_web_button_share_sheet&igsh=ZDNlZDc0MzIxNw==
Instituto Assaí	Assaí Atacadista + CUFA + FNA + Ação da Cidadania	Relatório anual e Instagram institucional	https://www.instagram.com/institutoassai/
Instituto C&A	Ação da Cidadania	Relatório anual, site e Instagram institucional	https://institutocea.org.br/ https://www.instagram.com/instituto_cea/
Instituto Clima e Sociedade	CIR + COIAB	Site e Instagram institucional	https://climaesociedade.org/ https://www.instagram.com/climaesociedade/
Instituto Sabin	CIR	Site e Instagram institucional	https://institutosabin.org.br/ https://www.instagram.com/instsabin/
Land is Life	COIAB	Site e Instagram institucional	https://www.landislife.org/news/ https://www.instagram.com/landislifeorg/
Rainforest Noruega	COIAB	Instagram institucional	https://www.regnskog.no/en/
Sesc	Ação da Cidadania + Global FoodBanking Network	Relatório anual e site institucional	https://www.sesc.com.br/ https://www.instagram.com/sescbrasil/
URIHI Associação Yanomami	CIR	Instagram institucional	https://www.instagram.com/urihiyanomami/
Visão Mundial	ADRA	Relatório anual, site e Instagram institucional	https://visaomundial.org.br/?fbclid=PAZXh0bgNhZW0CMTEAAaaHLNI_Vkd8ElcCGB0Ny22db53rwZ_usZYTlSgzGRCUL4zSDJ83tMdk05o_aem_eHnyg-lt47fI3N_sHPI0_Q https://www.instagram.com/visaomundialbr/
Ypassali Associação Sanuma	COIAB	Instagram	https://www.instagram.com/associacaosanuma?utm_source=ig_web_button_share_sheet&igsh=ZDNlZDc0MzIxNw==
ORGANISMOS INTERNACIONAIS			
Departamento de Proteção Civil e Ajuda Humanitária da União Europeia (Echo, acrônimo em inglês)	UNICEF Brasil	-	https://european-union.europa.eu/priorities-and-actions/actions-topic/humanitarian-aid-and-civil-protection_pt/
UNICEF Brasil	Água Camelo + ADRA + Echo + Fundação José Luiz Egydio Setúbal + OdontoPrev	Relatório anual, site e Instagram institucional	https://www.unicef.org/brazil/ https://www.instagram.com/unicefbrasil/

ACT-Brasil: Amazon Conservation Team; ADRA: Agência Adventista de Desenvolvimento e Recursos Assistenciais; Cepast/CNBB: Comissão episcopal pastoral para ação sociotrasnformadora da conferência nacional dos bispos; COIAB: Coordenação das Organizações Indígenas da Amazônia Brasileira; CIR: Conselho Indígena de Roraima; CUFA: Central Única das Favelas; CONDISI-YY: Conselho Distrital de Saúde Indígena Yanomami e Ye’kuana; DSEI: Distrito Sanitário Especial Indígena; Departamento de Proteção Civil e Ajuda Humanitária da União Europeia; FUNAI: Fundação Nacional dos Povos Indígenas; FNA: Frente Nacional Antirracista; iCS: Instituto Clima e Sociedade; MTST: Movimento dos Trabalhadores Sem-Teto; Sesc: Serviço Social do Comércio; SESAI: Secretaria de Saúde Indígena; UNICEF: Fundo das Nações Unidas para a Infância.

Fonte: elaboração própria.

Fonte: elaboração própria. Nota: a direção das setas indica a relação entre os diferentes atores. Para as setas que apresentam duas pontas, ambos os atores citaram as ações e parcerias desenvolvidas durante a emergência Yanomami. O ponto de partida da seta indica o ator que divulgou a informação. As logomarcas foram editadas para ocuparem o mesmo espaço na imagem.

A maioria (n = 27, 77,1%) das 35 ações mapeadas aconteceu por meio de interações entre estes atores, sendo os mais citados a Ação da Cidadania, a Central Única das Favelas (CUFA) e a CUFA Roraima. Somente seis realizaram alguma iniciativa isoladamente: Ação da Cidadania, Agência Adventista de Desenvolvimento e Recursos Assistenciais (ADRA), Cozinha Solidária do Movimento dos Trabalhadores sem Teto (MTST), Serviço Social do Comércio (Sesc), Real Cestas e UNICEF (Fundo das Nações Unidas para a Infância, Brasil). Os únicos atores mapeados que atuaram junto com associações indígenas foram o Conselho Indígena de Roraima e a Coordenação das Organizações Indígenas da Amazônia Brasileira (COIAB), que destinaram doações de alimentos em 2023.

Dentre as ações identificadas, a distribuição de alimentos (n = 16, 45,7%) e campanhas para arrecadar recursos financeiros (n = 8, 22,8%) foram as mais recorrentes. Entretanto, foram registradas doações de kits de filtração de água, bebedouros, água potável, envio de nutricionistas para atuar na emergência e editais para convocação de nutricionistas. Quase todas as ações (n = 32, 91,4%) aconteceram no ano de 2023, especialmente no primeiro trimestre.

Em 81,3% (n = 13) das doações não foram descritos quais gêneros alimentícios foram entregues. Em alguns casos, apesar de não haver descrição, pelas imagens de divulgação das ações foi possível identificar doações de arroz, feijão, açúcar, óleo e farinha de milho, mas também de alimentos ultraprocessados, como molhos de tomate, biscoitos salgados e biscoitos doces recheados. Quando houve descrição dos gêneros doados, não foram citados alimentos ultraprocessados, apesar de terem sido identificados na análise das imagens. As ações citadas estão sistematizadas no Quadro 3, no qual foram codificadas entre F1 e F27.

**Quadro 3:** Ações desenvolvidas pelos diferentes atores em resposta à situação de emergência nutricional do povo Yanomami no âmbito da alimentação e nutrição.

CÓDIGO	ATOR	AÇÃO REALIZADA	FONTE
F1	Coordenação das Organizações Indígenas da Amazônia Brasileira	Doação de alimentos em parceria com atores de organizações não governamentais	https://www.instagram.com/p/Cox3DRhpeHI/?utm_source=ig_web_copy_link&igsh=MzRlODBiNWFlZA%3D%3D&img_index=1
F2	Sociedade Esportiva Palmeiras	Divulgação da doação de alimentos para a CUFA	https://www.palmeiras.com.br/noticias/palmeiras-vai-arrecadar-alimentos-para-o-povo-yanomami-em-jogo-no-allianz-parque/
F3	Ação da Cidadania	Divulgação dos alimentos doados na emergência Yanomami	https://www.instagram.com/p/CoGOHu6JKt6/?utm_source=ig_web_copy_link&igsh=MzRlODBiNWFlZA%3D%3D
F4	Sesc	Divulgação dos alimentos doados na emergência Yanomami	https://www.sesc.com.br/noticias/assistencia/sesc-mesa-brasil-entrega-doacoes-ao-povo-indigena-yanomami/
F5	Sesc	Divulgação dos alimentos doados na emergência Yanomami	https://www.sesc.com.br/noticias/assistencia/sesc-faz-doacao-emergencial-aos-yanomami/
F6	Água Camelo	Divulgação da atuação no território Yanomami	https://aguacamelo.com.br/yanomami/
F7	Ação da Cidadania	Divulgação da atuação no território Yanomami e utilização do logo do iFood	https://uploads.strikinglycdn.com/files/6d349699-75cc-48b8-acbb-740419d2f0da/relatorio2023.pdf.
F8	iFood	Divulgação da doação de recursos para a CUFA	https://institucional.ifood.com.br/wp-content/uploads/2024/01/Ifood_info_transparencia_23_1Trimestre_.pdf
F9	Conselho Indígena de Roraima	Divulgação da doação de alimentos para os Yanomami	https://www.instagram.com/p/CvfQA_fupjz/?utm_source=ig_web_copy_link&igsh=MzRlODBiNWFlZA%3D%3D&img_index=1
F10	CUFA	Divulgação da campanha para arrecadar alimentos para a emergência Yanomami	https://cufa.org.br/cufa-e-frente-nacional-antirracista-lancam-campanha-para-ajudar-os-yanomami-e-regiao/
F11	CUFA Roraima	Divulgação da atuação no território Yanomami e parceria com a FavelaLLog	https://www.instagram.com/p/Cn7LADpJItX/?utm_source=ig_web_copy_link&igsh=MzRlODBiNWFlZA%3D%3D
F12	Real Cestas	Divulgação da parceria com a CUFA	https://www.instagram.com/reel/CoDLPDuJk7E/?utm_source=ig_web_copy_link&igsh=MzRlODBiNWFlZA==
F13	Real Cestas	Campanha para doação de cestas básicas para a emergência Yanomami	https://www.instagram.com/p/Cn7XOPIptmh/?utm_source=ig_web_copy_link&igsh=MzRlODBiNWFlZA==
F14	Ambev	Divulgação da atuação na emergência Yanomami	api.mziq.com/mzfilemanager/v2/d/c8182463-4b7e-408c-9d0f-42797662435e/ef00e5eb-ba41-50b4-e4ff-9d8a01ef0a07?origin=1
F15	Água Camelo	Divulgação da rede de parceiros atuantes na emergência Yanomami	https://www.instagram.com/p/C1ZqY-oJlR5/?utm_source=ig_web_copy_link&igsh=MzRlODBiNWFlZA%3D%3D
F16	Instituto Assaí	Divulgação da doação de cestas básicas	https://institutoassai.org.br/docs/PORT_RA2023_INSTITUTO_ASSAI_FINAL.pdf
F17	Assaí	Divulgação da doação de cestas básicas	https://api.mziq.com/mzfilemanager/v2/d/ec14f0ab-c5d4-4b12-a413-b6cc7475ed98/dfb26f50-422d-d56a-7e3e-8c7be9702047?origin=1
F18	Instituto C&A	Divulgação da doação de recursos financeiros para Ação da Cidadania	https://www.instagram.com/p/Cn5V-zYPBEe/?utm_source=ig_web_copy_link&igsh=MzRlODBiNWFlZA==
F19	Ação da Cidadania	Divulgação de rede parceira atuante na emergência nutricional no território Yanomami	https://www.instagram.com/p/CpC8kKrvhQm/?utm_source=ig_web_copy_link&igsh=MzRlODBiNWFlZA==
F20	UNICEF	Divulgação das ações realizadas no território Yanomami durante a emergência de saúde pública	https://www.unicef.org/brazil/apoio-a-resposta-humanitaria-na-terra-indigena-yanomami
F21	Instituto Pensi	Divulgação de doação de alimentos terapêuticos e fórmulas nutricionais	https://institutopensi.org.br/uma-pequena-ajuda-humanitaria-aos-yanomamis/
F22	UNICEF	Divulgação da parceria com OdontoPrev para produção de material educativo	https://www.unicef.org/brazil/comunicados-de-imprensa/iniciativa-do-unicef-para-enfrentamento-a-desnutricao-infantil-indigena-visa-impactar-30-mil-criancas-ate-2025
F23	UNICEF	Divulgação da implementação do centro de recuperação nutricional	https://www.unicef.org/brazil/historias/estou-orgulhosa-em-trabalhar-para-esta-grande-familia-yanomami
F24	UNICEF	Divulgação da implementação do centro de recuperação nutricional	https://www.unicef.org/brazil/historias/minha-missao-e-cuidar-sinto-os-yanomamis-como-se-fossem-o-meu-proprio-povo
F25	ADRA	Edital de convocação para atuar na emergência Yanomami no programa de água potável da UNICEF	https://files.adventistas.org/v2.adra.org.br/2024/09/18122459/RR-015-PROCESSO-SELETIVO-UNICEF-ADRA.pdf
F26	ADRA	Edital de convocação para nutricionista atuar na emergência Yanomami	https://files.adventistas.org/v2.adra.org.br/2024/02/01175943/RR-001-PROCESSO-SELETIVO-ADRA-UNICEF-2024.pdf
F27	ADRA	Divulgação de doação de água em parceria com UNICEF e Visão Mundial	https://www.instagram.com/p/CoXhck8O0lh/?utm_source=ig_web_copy_link&igsh=MzRlODBiNWFlZA%3D%3D

ADRA: Agência Adventista de Desenvolvimento e Recursos Assistenciais; CUFA: Central única das Favelas; Sesc: Serviço Social do Comércio; UNICEF: Fundo das Nações Unidas para a Infância.

Fonte: elaboração própria.

Nota: o código é composto pela letra F (fonte) e o número da linha ocupada.

Em relação ao respeito à cultura e tradições alimentares da população Yanomami, apenas duas ações, realizadas pelo Assaí atacadista e pela COIAB, anunciaram essa informação. Porém, não foram detalhados quais alimentos foram doados. Em uma de suas ações, a COIAB distribuiu biscoitos salgados ultraprocessados ao mesmo tempo em que descrevia a doação realizada como uma ação que respeita a cultura alimentar Yanomami (F1).

Apenas três atores descreveram os alimentos doados em alguma ação: Sesc, Ação da Cidadania e a Sociedade Esportiva Palmeiras (Palmeiras). O Palmeiras divulgou apenas os gêneros alimentícios referentes à segunda campanha de arrecadação e doação, sendo discriminados alimentos não perecíveis, como macarrão, feijão, arroz, café, açúcar, leite em pó e óleo. Todos os alimentos arrecadados foram doados para a CUFA (F2). A Ação da Cidadania divulgou a doação de arroz, sardinha em lata, farinha de milho, farinha d’água, leite integral em pó e sal. No entanto, não foi possível identificar se todos os envios de cestas básicas seguiram essa lista de alimentos (F3). A ação mobilizada pelo Sesc pautou-se na nota técnica do Ministério da Saúde e parte da doação realizada foi viabilizada pelo Programa Mesa Brasil em parceria com o Global FoodBanking Network (F4, F5).

Dentre os atores comerciais, destacam-se a Água Camelo e o iFood. Todas as ações realizadas pela Água Camelo ocorreram em interação com outros atores, sendo registradas interações com nove atores. A *startup* realizou doação de kits para tratamento da água desenvolvidos com tecnologia própria (F6). Já o iFood atuou em parceria com duas ONGs (CUFA e Ação da Cidadania) ^18^ na distribuição de cestas básicas (F7). A empresa arrecadou e destinou recursos financeiros por meio de doações realizadas pelos consumidores na sua própria plataforma de vendas, que tem lanches e pizzas como principais alimentos comercializados (F8).

As ONGs nacionais com maior número de interações diretas com outros atores foram a CUFA (n = 12) e o Conselho Indígena de Roraima (CIR) (n = 10). O CIR, em parceria com onze atores, realizou uma campanha para arrecadar recursos financeiros que foram destinados para a compra de alimentos e itens de higiene para a população Yanomami. Não foram especificados os alimentos doados, apesar da doação de caixas de frango congelado ter sido declarada (F9). Esta foi a única ação monitorada que identificou a participação de associações indígenas que já atuavam no território antes da declaração da ESPIN.

Destaca-se o envolvimento de ONGs em pautas não diretamente relacionadas ao tema da alimentação e nutrição, como no caso da CUFA, que arrecadou recursos financeiros para a compra de alimentos e contou com o apoio logístico da FavelaLLog para a distribuição dos gêneros alimentícios (F10, F11). Nessa ação foi possível identificar a doação das cestas básicas do ator comercial Real Cestas, que atuou em parceria com a CUFA nesta e em outras emergências nacionais (F12, F13). 

Outras iniciativas envolveram representantes do Governo Federal, como a interação entre Água Camelo, Ambev e CUFA com a Fundação Nacional dos Povos Indígenas (FUNAI) para distribuição de kits para o tratamento de água (F14). A *startup* Água Camelo divulgou no seu perfil do Instagram que suas ações foram viabilizadas por parcerias entre UNICEF, CUFA, Ambev e Coca-Cola (F15).

Quem mais se envolveu em iniciativas com interação com atores de governo foi a ONG Ação da Cidadania, que realizou reuniões com diferentes ministérios articulando ações para atuar na TI Yanomami. Além destas iniciativas, a ONG atuou com o Instituto Assaí na distribuição de cestas básicas para a população Yanomami (F16, F17) e firmou parceria com o Instituto C&A (F18). Em fevereiro de 2023, a ONG atuou em conjunto com a Cáritas Brasileira, o Sesc e as Cozinhas Solidárias do MTST (F19).

O organismo internacional mais citado foi o UNICEF, que atuou com o envio de profissionais para a reestruturação de unidades de saúde e envio de alimentos terapêuticos e de fórmulas nutricionais (F20). A Fundação José Luiz Egydio Setúbal e o Magazine Luiza doaram para o UNICEF aproximadamente cinco toneladas de alimentos terapêuticos e fórmulas nutricionais utilizados para o tratamento da desnutrição aguda no território Yanomami (F21). Em 2024, o UNICEF divulgou a parceria com a OdontoPrev para a elaboração de materiais educativos para o fortalecimento das políticas públicas e a realização de diagnósticos e tratamento da desnutrição na TI Yanomami (F22). Identificou-se a atuação da ADRA e do Departamento de Proteção Civil e Ajuda Humanitária (ECHO) junto ao UNICEF Brasil para a implementação dos centros de recuperação nutricional (F23, F24). A ADRA realizou editais e convocação de voluntários para atuarem na emergência, na doação de alimentos e água, assim como no envio de nutricionistas para trabalharem no território Yanomami (F25, F26, F27). Além disso, em parceria com o UNICEF e a Visão Mundial Brasil, doou e instalou quatro bebedouros nas unidades de saúde na TI Yanomami (F27).

## Discussão

As respostas à situação emergencial dos Yanomami abarcaram diferentes tipos de ações de alimentação e nutrição, caracterizadas por um amplo espectro de abrangência, implementadas por um perfil institucional diverso de atores e incluíram: doações de cestas básicas, de gêneros alimentícios, de produtos terapêuticos, de recursos financeiros, materiais e humanos, distribuição de filtros e equipamentos para tornar a água potável. Muitas iniciativas não detalharam em seus relatórios e nos seus meios de divulgação a descrição dos alimentos doados e/ou como o recurso financeiro captado foi utilizado.

As ações foram desenvolvidas por atores comerciais, ONGs e organismos internacionais, na maioria das vezes, em articulação entre si e com atores governamentais. Os diferentes atores buscaram se posicionar como “parceiros” nesse momento de crise humanitária, e algumas dessas ONGs são conhecidas por realizarem atividades em vários territórios e têm atuado no enfrentamento emergencial da fome no Brasil ^29^. Ao observar as parcerias destas com os atores comerciais, cabe problematizar a legitimidade que estas ONGs conferem a estes atores.

Lacy-Nichols et al. ^30^, ao analisarem a prática dos atores comerciais na perspectiva dos determinantes comerciais da saúde, indicam que pode haver dificuldades em estabelecer fronteiras na atuação de alguns atores. Isto é, uma instituição categorizada como do “terceiro setor” pode apresentar práticas e/ou atividades mais próximas ao setor comercial, como a realização de uma atividade para promover uma empresa e ampliar as suas vendas. Estas organizações podem ser quasi-comerciais (agindo como se fossem comerciais) ou podem ser filiadas aos atores comerciais (como algumas fundações e institutos) ^30^.

As parcerias entre ONGs e atores comerciais complexificam a diferenciação entre atuação social e interesses comerciais. Com a proximidade entre esses atores, a solidariedade, expressa em iniciativas populares que contribuem para a construção e o fortalecimento da cidadania, pode ser invisibilizada e capturada pela filantropia corporativa ^31^. Ações de gestão da reputação dos atores comerciais aumentam sua legitimidade e credibilidade, sendo, geralmente, parte integrante de todas as suas outras práticas ^7^. Embora seja reconhecido que alguns desses esforços possam ter efeitos positivos, muitas vezes eles contribuem mais para a criação de reputação do que para gerar benefícios para a sociedade.

Essas estratégias podem ser identificadas como *socialwashing*, uma forma do ator transmitir uma boa imagem e ganhar credibilidade da sociedade como um todo ^32^. Isso pode facilitar a aceitação e/ou a confiança da sociedade para outras práticas desses atores, contribuindo para legitimar discursos. Ações de *socialwashing* podem ser um exemplo de estratégia de atividade política corporativa, dentro do quadro dos determinantes comerciais da saúde. As atividades políticas corporativas ^32^ são recorrentes e foram encontradas em estudos realizados em outros momentos de crise, como na pandemia de COVID-19 ^33^ ^,^ ^34^.

No caso dos Yanomami, estas doações não priorizaram alimentos básicos oriundos da produção da agricultura indígena, nem a preservação e a valorização de hábitos e conhecimentos tradicionais inerentes de povos indígenas. Em sua maioria, desconsideraram o hábito e a cultura alimentar regional e tradicional desses povos e facilitaram o consumo de alimentos ultraprocessados. Além disso, houve descumprimento das recomendações da Organização Internacional do Trabalho, ratificadas pelo Brasil em 2019, que prevê a participação e cooperação dos povos interessados na adoção de medidas voltadas a aliviar as dificuldades que esses povos enfrentam, considerando que apenas uma ação declarou esse envolvimento ^35^.

Nesse contexto, as ações de filantropia corporativa podem possibilitar a promoção e a publicidade de suas marcas e seus produtos e a diminuição de críticas sobre os impactos negativos à saúde resultantes do seu consumo ^31^. No entanto, estes produtos são associados a problemas sociais, ambientais e de saúde, no contexto dos sistemas alimentares ^36^, desestruturando culturas tradicionais, reorientando as relações de posse e uso da terra e, consequentemente, impactando negativamente a soberania e a segurança alimentar nutricional dos povos Yanomami.

Expressando preocupação em relação à doação de alimentos ultraprocessados, a Aliança pela Alimentação Adequada e Saudável elaborou uma nota recomendando que as ações emergenciais de doações de alimentos fossem alinhadas à PNSAN, à PNAN e aos Guias Alimentares Brasileiros, destacando a necessidade de haver valorização da cultura alimentar e saberes dos Yanomami. A nota alertou para a proteção contra as estratégias de atividade política corporativa realizadas pelos atores comerciais e situações de conflitos de interesse ^37^.

Situações de emergência podem ser vistas por alguns atores como oportunidades para alavancar sua imagem e reputação ^33^ ^,^ ^34^. O forte envolvimento de atores comerciais de alimentos ultraprocessados em iniciativas de combate à desnutrição, por exemplo, oportuniza uma relação positiva das comunidades acometidas com esses atores, o que pode impactar positivamente a visão sobre as marcas de seus produtos ^7^. Além disso, também pode envolver interações com governos e unidades de saúde em nível local. Portanto, podem gerar situações de conflito de interesse, visto que o interesse dos atores comerciais pode interferir de forma indevida nos interesses primários de garantia da segurança alimentar e nutricional das comunidades afetadas ^7^.

Entretanto, em situações de emergência, essas preocupações são ainda menos valorizadas, abrindo-se espaço para que as interações aconteçam, podendo gerar situações em que os riscos superam os benefícios da ação. Por isso, a implementação de ferramentas para prevenção, mitigação e gerenciamento de conflito de interesse nos processos decisórios deveria ser considerada, em especial em contextos emergenciais ^31^. Um exemplo é a ferramenta de triagem para avaliar potenciais interações com atores não estatais, publicada pela Organização Pan-Americana da Saúde (OPAS) para identificar, prevenir e gerenciar potenciais conflitos de interesse nas políticas e programas de nutrição ^38^. O Fundo Nacional de Desenvolvimento da Educação (FNDE) publicou uma nota técnica, com base nesta ferramenta, no contexto da execução do Programa Nacional de Alimentação Escolar (PNAE) ^39^, entretanto, no Brasil ainda não há normativas ou recomendações que apoiem as decisões sobre essas interações em emergências e, no presente estudo, foram encontradas diversas interações entre os diferentes atores com integrantes do Governo Federal.

Cabe também considerar, especialmente no caso analisado, que a desnutrição aguda pode ser ocasionada por motivos diversos e repercutir em toda a família. Portanto, é fundamental que as ações desenvolvidas no âmbito da alimentação e nutrição sejam pautadas nos princípios da soberania e da segurança alimentar e nutricional ^20^ ^,^ ^21^. Foram entregues aos Yanomami fórmulas nutricionais (F100 e F75) e alimentos terapêuticos para tratamento da desnutrição, produzidos pelo grupo francês Nutriset, por meio de parceria entre o Governo do Brasil com o UNICEF ^40^. Consideradas dietas lácteas terapêuticas com adição de gordura vegetal, carboidratos, vitaminas e minerais, elas são alimentos ultraprocessados para fins especiais e sua aplicabilidade é emergencial ^41^. São necessárias soluções estruturais sustentáveis, de longo prazo, e que promovam a autonomia dos povos ^42^ ^,^ ^43^.

Estudos realizados entre os grupos indígenas Yanomami relataram que eles experimentam um ciclo intergeracional contínuo de desnutrição ^44^ ^,^ ^45^, e que suas famílias têm um alto grau de vulnerabilidade socioambiental, o que resulta em um estado permanente de insegurança alimentar ^46^. Ainda que a desnutrição seja um dos problemas mais graves enfrentados na TI Yanomami, ela se insere no contexto sistêmico da sindemia global ^23^. Portanto, as respostas devem ser formuladas numa perspectiva igualmente sistêmica. Mesmo que doações pontuais de alimentos respondam à crise imediata, não resolvem a questão em longo prazo e geralmente não envolvem os Yanomami na busca por soluções, que demandam lidar com o garimpo ilegal e a iniquidade histórica a que essa população é exposta ^47^. Ademais, quando a doação é de alimentos ultraprocessados, sob o argumento de amenizar uma situação aguda de fome, ela pode contribuir para, em médio e longo prazos, fortalecer assimetrias de poder, problemas sociais, ambientais e de saúde no contexto dos sistemas alimentares ^7^ ^,^ ^36^.

Por se tratar de uma situação relativamente recente, uma das limitações do presente estudo foi a impossibilidade de analisar ações de 2024 de muitos atores comerciais, visto que seus relatórios anuais ainda não haviam sido publicados no momento da coleta de dados. Para minimizar essa limitação, procurou-se, quando possível, incluir relatórios parciais de 2024 nas análises. Outra limitação foi a não avaliação do impacto das doações sobre o estado nutricional e a saúde dos Yanomami, tema que merece ser investigado em estudos futuros.

## Conclusões

Foram observadas conexões entre diferentes segmentos de atores (ONGs, atores comerciais e setores de governo), mobilizados por uma situação emergencial. Essa rede evidencia, inicialmente, uma diversidade de inserções das instituições envolvidas no processo, considerando os interesses primários de cada uma e os possíveis fatores que podem ter motivado essa inserção. No caso dos atores comerciais, as diferentes formas de atividade política corporativa são mais evidentes e os resultados indicam pontos críticos associados com ações de gestão da reputação e *socialwashing* impulsionadas por esses atores. A conexão de organizações da sociedade civil com esses processos pode ser mobilizada por interesses políticos e econômicos. Ainda assim, não há como desconsiderar a vocação de atuação social e de interesse público de diversas organizações aqui identificadas.

Destaca-se a diversidade de respostas, incluindo diferentes tipos de serviços e doações de alimentos, muitas vezes não especificadas e, em alguns casos, indicadas como adequadas aos princípios das políticas públicas aqui referenciadas. Cabe considerar os riscos de médio e longo prazos associados à doação de alimentos ultrapocessados e valorizar os posicionamentos de ONGs que alertam para os riscos de captura política desses processos por meio de estratégias de atividade política corporativa e de situações de conflitos de interesse.

A existência de instrumentos destinados a gerenciar, prevenir e mitigar os danos dos determinantes comerciais da saúde, as situações de conflitos de interesse e a evidenciar como a atividade política corporativa se configura nesses contextos e em situações específicas contribui para minimizar esses riscos. Esses instrumentos podem também orientar os atores envolvidos na implementação de respostas que efetivamente garantam a segurança alimentar e nutricional, a soberania alimentar e o DHAA desses povos em curto, médio e longo prazos.

## Data Availability

As fontes de informação utilizadas no estudo estão indicadas no corpo do artigo.

## Material Suplementar

Material Suplementar
