# Supplementary material for: Nutritional emergency in the Yanomami territory: actions developed to combat hunger and the interface with commercial determinants of health
Source: Cad Saude Publica. 2025 Dec 1;41(11):e00073525. [Article in Portuguese] doi: 10.1590/0102-311XPT073525 (PMC12688209; doi:10.1590/0102-311XPT073525)
Supplement: Material Suplementar [file 1678-4464-csp-41-11-PT073525-s.pdf]

Material Suplementar

Quadro S1 Descrição dos atores identificados no estudo.

ATOR	LOGOMARCA	DESCRIÇÃO	FONTE DE DADOS *
**ATORES COMERCIAIS**			
Água camelo		Startup de impacto socioambiental que fornece água potável para onde for preciso por meio de tecnologias	Site (https://aguacamelo.com.br/quem-somos/)
Ambev		Empresa de capital aberto, sediada em São Paulo, com operações industriais ou comerciais em todo o Brasil e em diversos países das Américas. Possui 30 cervejarias, maltarias, refrigeranterias, fábrica de rótulos, rolha e vidro e mais de 100 centros de distribuição	Site (https://www.ambev.com.br/sobre-ambev)
Assaí atacadista		Empresa brasileira de atacarejo que atende pequenos(as) e médios comerciantes e consumidores(as)	Site (https://ri.assai.com.br/o-assai/quem-somos/)
Band Tv		É uma rede de televisão comercial aberta brasileira, pertencente ao Grupo Bandeirantes	Wikipédia (https://pt.wikipedia.org/wiki/Band)
Coca-Cola		Empresa de bebidas não alcoólicas que abarca mais de 200 marcas e está presente em mais de 200 países e territórios	Site (https://www.coca-cola.com/br/pt/about-us/nos-conheca)
CVS Cestas Básicas		Empresa existente há mais de 20 anos que busca oferecer soluções funcionais e inovadoras para empresas através do fornecimento de cestas básicas e do cartão multibenefícios	Site (https://cvscesta.com.br/sobre/)
Favela LLog		Empresa que realiza entregas e distribui produtos em favelas e periferias de todo o Brasil	Site (https://favelallog.com.br/)
Ford		Fabricante de automóveis da multinacional estadunidense	Wikipédia (https://pt.wikipedia.org/wiki/Ford_Motor_Company)

iFood		Empresa brasileira de tecnologia atuando com delivery por meio de plataforma digital	Site (https://institucional.ifood.com.br/noticias/o-que-e-o-ifood/)
Magazine Luiza		Desde 2019 atua como uma plataforma digital de varejo com venda de produtos diversos, como móveis, eletrodomésticos, eletroportáteis, suplementos alimentares, bebidas e utilidades domésticas	Site (https://ri.magazineluiza.com.br/show.aspx?idCanal=urUqu4hANldyCLgMRgOsTw==)
Mercado Pago		Banco digital brasileiro	Instagram (https://www.instagram.com/mercadopagobr/)
OdontoPrev		Plataforma de saúde bucal com oferta de planos odontológicos	Site (https://www.odontoprev.com.br/quem-somos/nossa-estrutura)
Sociedade Esportiva Palmeiras		Sociedade esportiva fundada em 1914	Site (https://www.palmeiras.com.br/instituicao/)
Real Cestas		Atuam oferecendo soluções corporativas por meio de cestas básicas, de Natal e personalizadas	Site (https://www.realcestas.com.br/quem-somos/)
**Atores de organizações nacionais e internacionais não governamentais**			
Ação da Cidadania		Organização da sociedade civil que visa mobilizar a sociedade para erradicar a fome e a desigualdade com promoção do acesso à alimentação adequada e construindo uma sociedade mais justa e solidária	Site (https://www.acaodacidadania.org.br/documentos-and-prestacao-de-conta)
Agência Adventista de Desenvolvimento e Recursos Assistenciais (Adra)		Agência humanitária da Igreja Adventista do Sétimo Dia presente em mais de 130 países. No Brasil, a ADRA está organizada em 16 regionais que atendem todos os estados brasileiros	Site (https://adra.org.br/nossa-equipe/)
Amazon Conservation Team (ACT-Brasil)		Organização socioambiental sem fins lucrativos que atua pela conservação dos ecossistemas tropicais na América do Sul. No Brasil, atua principalmente na região Amazônica	Site (https://brasil.amazonteam.org/quem-somos-2/)

Associação Hutukara Yanomami		Organização indígena com papel de articulação política e de gestão de projetos voltados à proteção territorial, etnoambiental, saúde, formação, pesquisa e outras iniciativas	Site e Instagram institucional (https://hutukarayanomami.org/hutukara/)
Cáritas Brasileira		Organismo da Conferência Nacional dos Bispos do Brasil (CNBB) fundada em 1956 originada da ação mobilizadora de Dom Helder Câmara. Conta com uma rede com 198 entidades-membros, 13 regionais e 4 articulações	Site (https://caritas.org.br/historia)
Comissão episcopal pastoral para ação sociotransformadora da conferência nacional dos bispos (Cepast - CNBB)		Comissão que atua para fortalecer a participação da Igreja na formação do desenvolvimento humano integral para a construção de uma sociedade justa e solidária	Site (https://cepastcnbb.org.br/)
Conselho Indígena de Roraima (CIR)		Organização indígena de base fundada em 1970 a partir da realização da primeira Assembléia dos Tuxauas	Site (https://www.cir.org.br/post/cir)
Coordenação das Organizações Indígenas da Amazônia Brasileira (COIAB)		Organização indígena fundada em 1989. Surgiu da articulação de lideranças de organizações indígenas existentes visando uma auto representação na luta pelos direitos	Site (https://coiab.org.br/a-coiab/nossa-luta/)
Cozinha Solidária do Movimento dos Trabalhadores Sem-Teto (MTST)		Movimento social que nasceu em 1997 com o intuito de garantir o direito constitucional à moradia digna para todas e todos. As cozinhas solidárias foram criadas no período da pandemia do COVID-19 visando garantir alimentação diária e gratuita para famílias periféricas	Site (https://mtst.org/quem-somos/o-mtst e https://mtst.org/cozinhas-solidarias/)
Central Única de Favelas (CUFA)		Organização brasileira presente há mais de 25 anos nas favelas brasileiras que promove inclusão social por meio de esportes, cultura, educação, cidadania e empreendedorismo	Site (https://cufa.org.br/quem-somos/)
CUFA Roraima		Regional da CUFA situada em Roraima	Instagram (https://www.instagram.com/cufa_rr/)

Diocese de Roraima		Sede episcopal que busca apoiar e fornecer suporte à população migrante e indígena presentes no estado junto com a missão de evangelização	Site (https://diocesederoraima.org.br/sobre/)
Frente Nacional Antirracista (FNA)		Frente que atua na promoção da ampla participação da comunidade negra no debate político e econômico brasileiro	Instagram (https://www.instagram.com/frentenacionalantirracista/)
Fundação José Luiz Egydio Setúbal – Instituto Pensi		Fundação criada por José Luiz Egydio Setúbal com atuação múltipla tendo como temas principais: saúde mental, imunização e segurança alimentar. O Instituto Pensi busca desenvolver pesquisas e tecnologias alternativas além de produzir, divulgar e ensinar conhecimentos técnicos e científicos nas áreas de saúde infanto-juvenil	Site (https://fundacaojles.org.br/quem-somos/historico/ e https://fundacaojles.org.br/nosso-trabalho/instituto-pensi/)
Fundo Brasil		Fundação independente, sem fins lucrativos, criada por atividades para fortalecer organizações da sociedade civil na luta por direitos humanos	Site (https://www.fundobrasil.org.br/conheca-o-fundo-brasil/)
Global FoodBanking Network		Rede que atua com parceiros em mais de 50 países para recuperar e redirecionar alimentos para aqueles que precisam por meio de bancos de alimentos	Site (https://www.foodbanking.org/about-gfn/)
Instituto A Nossa Jornada		Organização não governamental fundada por Renata Quintella	Instagram institucional (https://www.instagram.com/institutoanossajornada)
Instituto Assaí		O Instituto Assaí é uma organização independente e sem fins lucrativos, responsável por todas as iniciativas de investimento social do Assaí Atacadista	Site (https://institutoassai.org.br/quem-somos/)
Instituto C&A		Pilar social da C&A Brasil. Desenvolve ações voluntárias, projeto de fomento à inclusão produtiva com apoio ao empreendedorismo, à empregabilidade e à trabalhabilidade no setor da moda	Site (https://institutocea.org.br/sobre-nos/quem-somos/)
Instituto Clima e Sociedade		Organização filantrópica que apoia o enfrentamento das mudanças climáticas no Brasil	Site (https://climaesociedade.org/sobre/)
Instituto Sabin		Organização da Sociedade Civil de Interesse Público (OSCIP), para melhoria da qualidade de vida, do bem-estar e da prosperidade nas comunidades onde o Grupo Sabin atua, fomentando a inovação social	Site (https://institutosabin.org.br/)

Land is Life		Coalizão global de povos indígenas que trabalha para promover a autodeterminação e os direitos coletivos dos povos indígenas	Site (https://www.landislife.org/about-us/#mission)
Rainforest Noruega		Organização não governamental internacional que atua para proteger as florestas tropicais e os direitos dos povos indígenas e das florestas por meio de uma rede com parceiros locais	Site (https://www.regnskog.no/en/what-we-do/about-us)
Sesc (Serviço Social do Comércio)		Criado em 1946 como compromisso de que empresários do setor coloboraríam com o cenário social por meio de ações que beneficiassem empregados e seus familiares. Com o tempo essas ações foram voltadas para toda a população	Site (https://www.sesc.com.br/institucional/o-sesc/sesc/)
URIHI Associação Yanomami		Organização não governamental	Instagram institucional (https://www.instagram.com/urihiyanomami/)
Visão Mundial		Organização não-governamental cristã internacional que trabalha em 100 países em todo o mundo. No Brasil, atua com foco nas crianças e adolescentes em situação de maior vulnerabilidade	Site (https://visaomundial.org.br/nossa-historia)
Ypassali Associação Sanuma		Organização não governamental	Instagram institucional (https://www.instagram.com/associacaosanuma?utm_source=ig_web_button_share_sheet&igsh=ZDNlZDc0MzIxNw==)
**Organismos internacionais**			
Departamento de Proteção Civil e Ajuda Humanitária da União Europeia (Echo, na sigla em inglês)		Departamento da União Europeia criado em 1992 que fornece assistência humanitária	Site (https://civil-protection-humanitarian-aid.ec.europa.eu/who/about-echo_en)

UNICEF Brasil		Organismo internacional que trabalha pela garantia dos direitos da criança e do adolescente e que desde 1950 apoia transformações na área da infância e da adolescência no Brasil	Site (https://www.unicef.org/brazil/o-que-fazemos)

* Os links foram acessados em 26 de março de 2025.
Fonte: Elaborado pelas autoras.

Quadro S2 Síntese das ações desenvolvidas pelos diferentes atores em resposta à situação de emergência nutricional do povo Yanomami no âmbito da alimentação e nutrição (2023 e 2024).

ATOR(ES) ENVOLVIDO(S)	AÇÃO REALIZADA	DESCRIÇÃO	ANO DA AÇÃO	FONTE DE DADOS
Ação da Cidadania<br>+ deputado federal Alessandro Molon<br>+Ministro do desenvolvimento agrário e Familiar<br>+Ministro do desenvolvimento e assistência social, família e combate à fome<br>+Ministra dos povos indígenas, Funai<br>+ Militares	Reuniões de articulação com governo federal para atuar na agenda da fome	Não houve divulgação das pautas e encaminhamentos de todas as reuniões que ocorreram	2023	Instagram
Ação da cidadania<br>+ iFood	Doação de cestas básicas	Não foram divulgadas composições nem a quantidade de cestas doadas	2023	Relatório anual da Ação da Cidadania
Ação da Cidadania<br>+ Instituto C&A	Doação de 50 mil reais em cestas básicas do Instituto C&A para a Ação da Cidadania	Não foram divulgadas composições das cestas básicas	2023	Instagram Ação da Cidadania e Instituto C&A, Site e relatório Instituto C&A
Ação da Cidadania<br>+ Sesc − Mesa Brasil<br>+ Cáritas Brasileira<br>+ Cozinha Solidária do MTST	Atuação conjunta no território para sanar a fome do povo Yanomami	Não foi descrito a forma de atuação	2023	Instagram Ação da Cidadania
Assaí atacadista<br>+ CUFA<br>+ CUFA Roraima<br>+ Ação da cidadania<br>+ FNA	Doação de cestas básicas realizada pelo Instituto Assaí	Doação de 12 toneladas de alimentos - mil cestas básicas, respeitando o cardápio dos Yanomami. Não foi divulgado quais alimentos foram doados	2023	Relatório anual do Assaí Atacadista, Instagram CUFA e CUFA Roraima
Ambev<br>+ Água Camelo<br>+ CUFA<br>+ CUFA Roraima<br>+ FNA<br>+ FUNAI	Distribuição de kits para o tratamento de água	Foram doados 180 kits para filtrar água. Os kits são estruturados dentro de mochilas, com capacidade de armazenar até 15 litros	2023	Relatório anual da Ambev, site institucional da Água Camelo, instagram Água Camelo, CUFA, CUFA Roraima e FNA
Água Camelo<br>+ UNICEF<br>+ CUFA<br>+ Ambev<br>+ Coca-Cola<br>+ FINAI<br>+ SESAI<br>+ DSEI Yanomami	Distribuição de kits para o tratamento de água	Não foram divulgados quantos kits foram doados nesta parceria	2023	Instagram Água Camelo
ADRA<br>+ UNICEF	Editais para seleção de profissionais para atuar na emergência Yanomami	Seleção de profissionais para atuar no projeto WASH e no território Yanomami	2024	Site institucional
Adra<br>+ UNICEF Brasil<br>+ Visão Mundial Brasil	Doação de bebedouros para unidades de saúde onde aconteceram os atendimentos para os Yanomami	Doação de 4 bebedouros para as unidades de saúde	2023	Instagram ADRA Brasil
CUFA<br>+ CUFA Roraima<br>+ FNA<br>+ Favela LLog<br>+ iFood<br>+ Band	Arrecadação de dinheiro, via plataforma online, para a compra e distribuição de alimentos	Não foi divulgado o valor total arrecadado nem quais alimentos foram doados	2023	Site institucional da CUFA, Instagram CUFA, CUFA Roraima, FNA

CUFA<br>+ CUFA Roraima<br>+ FNA<br>+ Favela LLog	Arrecadação de dinheiro e doação de cestas básicas	Não foi divulgado o valor total arrecadado nem quais alimentos foram doados	2023	Instagram CUFA, CUFA Roraima
CUFA<br>+ CUFA Roraima<br>+ FUNAI	Doação de cestas básicas e distribuição para as casas de apoio aos indígenas	Envio das cestas doadas para a Funai realizar a distribuição. Não foram divulgadas informações sobre a composição das cestas	2023	Instagram CUFA e CUFA Roraima
Cáritas Brasileira<br>+ CIR<br>+ Cepast/CNBB<br>+ Diocese de Roraima	Campanha para arrecadação de dinheiro para compra de alimentos	Não houve divulgação dos valores arrecadado	2023	Instagram Cáritas Brasileira
CIR<br>+CONDISI-YY<br>+ URIHI Associação Yanomami<br>+ COIAB<br>+ Fundo Brasil<br>+ Instituto Sabin<br>+ Instituto A Nossa Jornada<br>+ Ford<br>+ iCS	Arrecadação e distribuição de alimentos na campanha SOS Yanomami	Divulgação parcial das doações, sendo 110 cestas básicas e 11 caixas de frango Não foram divulgados quais alimentos foram doados na cesta básica	2023	Site institucional CIR
COIAB<br>+ *Amazon Conservation Team* (ACT-Brasil)<br>+ *Rainforest Noruega*<br>+ *Land is Life*<br>+ Associação Hutukara Yanomami<br>+ CIR	Doação de cestas básicas	Doação de 6 toneladas de alimentos<br>É indicado que os alimentos respeitam a cultura alimentar, mas não são divulgados os alimentos doados	2023	Instagram COIAB
COIAB<br>+ CIR<br>+ Hutukara associação Yanomami<br>+ Ypassali Associação Sanuma	Arrecadação de dinheiro para reverter em alimentos	Não foram divulgadas informações sobre valores e alimentos doados	2023	Instagram COIAB
iFood<br>+ CUFA	Doação de cestas básicas	Foram doadas 800 cestas, não sendo disponibilizadas informações sobre a composição das cestas básicas	2023	Relatório institucional do iFood
iFood<br>+ CUFA	Arrecadação de dinheiro via aplicativo do iFood para doação para a CUFA	Foi arrecadado R$ 501.441,00 entre 21/01- 15/02/2023	2023	Relatório institucional do iFood
Instituto a nossa jornada<br>+ DSEI Roraima<br>+ Funai<br>+ CIR	Doação de cestas básicas	Não foi divulgado quais alimentos foram doados	2023	Instagram Instituto a nossa jornada
Palmeiras<br>+ CUFA	Arrecadação de alimentos nos jogos do campeonato paulista e copa São Paulo de futebol júnior e doação para a CUFA	Doação de 3 toneladas de alimentos não perecíveis, sendo macarrão, feijão, arroz, café, açúcar, leite em pó e óleo	2023	Site institucional e Instagram Palmeiras, Instagram CUFA
Sesc - Mesa Brasil<br>+ *Global FoodBanking Network*	Doação de dinheiro do Global FoodBanking Network para o Sesc	Doação de 250 milhões de reais para aquisição de cestas básicas, com alimentos de acordo com a nota técnica nº 1/2023/COASI/CGPDS/DPDS-FUNAI	2023	Site institucional do Sesc
Real Cestas<br>+ CUFA	Doação de cestas básicas junto com a CUFA	Não foi divulgado o valor total arrecadado nem quais alimentos foram doados	2023	Instagram Real Cestas
UNICEF Brasil	Doação de dieta terapêutica a base	Doação de duas toneladas de	2024	Site institucional

+ Fundação José Luiz Egydio Setúbal<br>+ Magazine Luiza	de leite, F-75 e F-100, ReSoMal e micronutriente em pó	ReSoMal, 0,3 tonelada de leite terapêutico F75, cinco toneladas de leite terapêutico F100 e três toneladas de micronutrientes em pó para prevenção à desnutrição		UNICEF e site institucional da Fundação José Luiz Egydio Setúbal
UNICEF Brasil<br>+ Fundação José Luiz Egydio Setúbal	Doação de suplemento alimentar por meio de sachês nutricionais	Não foi divulgado o total doado nem quais suplemento foi utilizado	2023	Site institucional UNICEF e site institucional da Fundação José Luiz Egydio Setúbal
UNICEF Brasil<br>+ SESAI<br>+ DSEI Yanomami<br>+ Governo Federal (Ministra dos povos indígenas, Ministério da Saúde)	Articulação com o governo federal para distribuição de medicamentos e insumos para tratamento de desnutrição aguda, ações voltadas a água, saneamento e higiene	Não foram divulgados dados sobre as reuniões e articulações.	2023	Site institucional UNICEF Brasil
UNICEF Brasil<br>+ ADRA<br>+ Echo	Implementação da alimentação terapêutica	Os atores implementaram em parceria com o Unicef os centros de tratamento para desnutrição aguda. Não foram divulgadas mais informações	2023	Site institucional UNICEF
UNICEF<br>+ OdontoPrev	Capacitação profissional e criação de cartilhas para identificação e tratamento da desnutrição	Não foram divulgados dados da ação	2024	Site institucional UNICEF
**Ações desenvolvidas por apenas um ator**				
Ação da Cidadania	Doação de alimentos e cestas básicas e arrecadação de dinheiro	Divulgação parcial dos alimentos e quantidades doados, em 31/01/2023. Lista de alimentos: Arroz, latas de sardinha, farinha de milho, farinha d'água, leite integral em pó e sal	2023	Instagram
ADRA	Disponibilizou duas nutricionistas para atuar na emergência Yanomami	Duas nutricionistas para atuar na CASAI	2023	Instagram
ADRA	Campanha para arrecadação de fundos para compra de alimentos e água	Não foram divulgados os alimentos nem a quantidade doadas	2023	Instagram
Cozinha solidária MTST	Arrecadação de alimentos e fundos para destinar aos Yanomami	Não foram divulgados os alimentos nem a quantidade doadas	2023	Instagram
Sesc	Doação de cestas básicas em prol dos Yanomami	O Sesc informou que as cestas continham os alimentos orientados pela nota técnica nº 1/2023/COASI/CGPDS/DPDS-FUNAI e foram entregues 5.000 cestas básicas	2023	Site e instagram institucional do Sesc
Real Cestas	Campanha para doação de cestas para os Yanomami	Não foi divulgado o total doado nem quais alimentos foram doados	2023	Instagram Real Cestas
UNICEF Brasil	Doação de Soro de Reidratação Oral para Crianças com Desnutrição Grave (RESOMAL)	Foram doadas 36.000 sachês. O produto apresenta a seguinte composição: Glicose 125mmol/L, Sódio 45mmol/L, Potássio 40mmol/L, Cloreto 70mmol/L, Citrato 7mmol/L, Magnésio 3mmol/L, Zinco 0,3mmol/L, Cobre 0,045mmol/L, Osmolaridade da solução preparada: 300mmol/L	2023	Site institucional

	Aquisição contínua de 240 latas de leite<br>terapêutico F75 e 9.600 de leite terapêutico F100	Foram doados 10 caixas de dieta F-75 (com cada caixa contendo 24 latas de 400g) e 400 caixas de dieta F-100 (com cada caixa contendo 24 latas de 400g)<br><br>As dietas são compostas feitas a base de leite com adição de gordura vegetal, carboidratos, vitaminas e minerais	2023	Site institucional

ADRA: Agência Adventista de Desenvolvimento e Recursos Assistenciais; CASAI: Casa de apoio à saúde indígena nacional; Cepast/CNBB: Comissão episcopal pastoral para ação sociotrasnformadora da conferência nacional dos bispos; CIR - Conselho Indígena de Roraima; COIAB: Coordenação das Organizações Indígenas da Amazônia Brasileira; CONDISI-YY: Conselho Distrital de Saúde Indígena Yanomami, Yekuana; CUFA: Central Única das Favelas; CUFA Roraima: Central Única das Favelas Roraima; DSEI Y: Distrito Sanitário Especial Indígena Yanomami; Echo: Departamento de Proteção Civil e Ajuda Humanitária; iCS: Instituto Clima e Sociedade, FNA: Frente Nacional Antirracista, Funai: Fundação Nacional do Povos Indígenas; MTST: Movimento dos Trabalhadores Sem-Teto, Sesc Serviço social do comércio; SESAI: Secretaria de Saúde indígena; UNICEF: Fundo das Nações Unidas para a Infância.

Fonte: elaborado pelos autores.

Quadro S3 Dados coletados sobre a atuação de atores comerciais, organizações não governamentais e organismos internacionais na emergência nutricional na terra indígena Yanomami nos anos de 2023 e 2024.

ATOR	DATA DA PUBLICAÇÃO	CONTEÚDO	WEBSITE	ÚLTIMO ACESSO
Ação da Cidadania	25/01/2024	Post sobre as doações de alimentos feitas no ano de 2023 com menção a ação no território Yanomami	https://www.instagram.com/p/C2h2diYLt1T/?utm_source=ig_web_copy_link&igsh=MzRlODBiNWFlZA==	21/03/2025
Ação da Cidadania	30/12/2023	Post sobre as doações de alimentos feitas no ano de 2023 com menção a ação no território Yanomami	https://www.instagram.com/p/C1emPl6PaPs/?utm_source=ig_web_copy_link&igsh=MzRlODBiNWFlZA==	21/03/2025
Ação da Cidadania	27/12/2023	Post sobre as doações de alimentos feitas no ano de 2023 com menção a ação no território Yanomami	https://www.instagram.com/p/C1W34nFqeUZ/?utm_source=ig_web_copy_link&igsh=MzRlODBiNWFlZA==	21/03/2025
Ação da Cidadania	15/06/2023	Post referente ao encontro com a Ministra dos povos indígenas, pautando as ações desenvolvidas no território Yanomami	https://www.instagram.com/p/Cth1b98PyZ8/?utm_source=ig_web_copy_link&igsh=MzRlODBiNWFlZA==	21/03/2025
Ação da Cidadania	19/04/2023	Post sobre doação de alimentos para os Yanomami	https://www.instagram.com/p/CrOJNDesQaA/?utm_source=ig_web_copy_link&igsh=MzRlODBiNWFlZA==	21/03/2025
Ação da Cidadania	15/04/2023	Post sobre doação de alimentos para os Yanomami	https://www.instagram.com/p/CrDsSk0NF3d/?utm_source=ig_web_copy_link&igsh=MzRlODBiNWFlZA==	21/03/2025
Ação da Cidadania	10/04/2023	Post sobre ação para arrecadar alimentos para os Yanomami	https://www.instagram.com/reel/Cq3ZqsApf3E/?utm_source=ig_web_copy_link&igsh=MzRlODBiNWFlZA==	21/03/2025
Ação da Cidadania	08/03/2023	Post sobre doação de alimentos para os Yanomami	https://www.instagram.com/p/CpizsFtJZ2H/?utm_source=ig_web_copy_link&igsh=MzRlODBiNWFlZA==	21/03/2025
Ação da Cidadania	24/02/2023	Post sobre ações realizadas no território Yanomami e rede de parceiros	https://www.instagram.com/p/CpC8kKrvhQm/?utm_source=ig_web_copy_link&igsh=MzRlODBiNWFlZA==	21/03/2025
Ação da Cidadania	09/02/2023	Post sobre ações realizadas no território Yanomami e rede de parceiros	https://www.instagram.com/reel/CocoHTxNunr/?utm_source=ig_web_copy_link&igsh=MzRlODBiNWFlZA==	21/03/2025
Ação da Cidadania	06/02/2023	Post divulgando a doação de alimentos através de matéria jornalística	https://www.instagram.com/reel/CoVq9OTvAy-/?utm_source=ig_web_copy_link&igsh=MzRlODBiNWFlZA==	21/03/2025
Ação da Cidadania	03/02/2023	Post divulgando a campanha de arrecadação de dinheiro	https://www.instagram.com/p/CoNSRo7JV1d/?utm_source=ig_web_copy_link&igsh=MzRlODBiNWFlZA==	21/03/2025
Ação da Cidadania	01/02/2023	Post divulgando a doação de alimentos através de matéria jornalística	https://www.instagram.com/reel/CoI6R-Usq-Y/?utm_source=ig_web_copy_link&igsh=MzRlODBiNWFlZA==	21/03/2025
Ação da Cidadania	31/01/2023	Post divulgando a doação de alimentos através de matéria jornalística	https://www.instagram.com/p/CoGOHu6JKt6/?utm_source=ig_web_copy_link&igsh=MzRlODBiNWFlZA==	21/03/2025
Ação da Cidadania	30/01/2023	Post divulgando a campanha de arrecadação de dinheiro e dos alimentos doados	https://www.instagram.com/reel/CoDvwgnt88M/?utm_source=ig_web_copy_link&igsh=MzRlODBiNWFlZA==	21/03/2025
Ação da Cidadania	30/01/2023	Post divulgando a campanha de arrecadação de dinheiro	https://www.instagram.com/p/CoDLm7UIqjh/?utm_source=ig_web_copy_link&igsh=MzRlODBiNWFlZA==	21/03/2025
Ação da Cidadania	29/01/2023	Post divulgando a campanha de arrecadação	https://www.instagram.com/reel/CoALaJQNkKi/?	21/03/2025

		de dinheiro com atores brasileiros	utm_source=ig_web_copy_link&igsh=MzRlODBiNWFlZA==	
Ação da Cidadania	28/01/2023	Post divulgando a campanha de arrecadação de dinheiro	https://www.instagram.com/reel/Cn-LO6yplmY/?utm_source=ig_web_copy_link&igsh=MzRlODBiNWFlZA==	21/03/2025
Ação da Cidadania	26/01/2023	Post divulgando a doação de alimentos e campanha para arrecadar recursos através de matéria jornalística	https://www.instagram.com/reel/Cn5ZrnFsGTU/?utm_source=ig_web_copy_link&igsh=MzRlODBiNWFlZA==	21/03/2025
Ação da Cidadania	26/01/2023	Post divulgando a doação de cestas básicas em parceria com o Instituto C&A	https://www.instagram.com/p/Cn5V-zYPBEe/?utm_source=ig_web_copy_link&igsh=MzRlODBiNWFlZA==	21/03/2025
Ação da Cidadania	26/01/2023	Post sobre reunião com diferentes membros do governo para mobilizar ações no território Yanomami	https://www.instagram.com/p/Cn4-9l1SBLJ/?utm_source=ig_web_copy_link&igsh=MzRlODBiNWFlZA==	21/03/2025
Ação da Cidadania	26/01/2023	Post divulgando a doação de alimentos e campanha para arrecadar recursos através de matéria jornalística	https://www.instagram.com/reel/Cn4kOQKoYa3/?utm_source=ig_web_copy_link&igsh=MzRlODBiNWFlZA==	21/03/2025
Ação da Cidadania	25/01/2023	Post divulgando a doação de alimentos e campanha para arrecadar recursos	https://www.instagram.com/reel/Cn2yKvSJZQq/?utm_source=ig_web_copy_link&igsh=MzRlODBiNWFlZA==	21/03/2025
Ação da Cidadania	25/01/2023	Post divulgando a doação de alimentos e campanha para arrecadar recursos através de matéria jornalística	https://www.instagram.com/reel/Cn2drvWuYrh/?utm_source=ig_web_copy_link&igsh=MzRlODBiNWFlZA==	21/03/2025
Ação da Cidadania	25/01/2023	Post sobre reunião com diferentes membros do governo para mobilizar ações no território Yanomami	https://www.instagram.com/p/Cn2Iho7JPRM/?utm_source=ig_web_copy_link&igsh=MzRlODBiNWFlZA==	21/03/2025
Ação da Cidadania	24/01/2023	Post divulgando a doação de alimentos e campanha para arrecadar recursos através de matéria jornalística	https://www.instagram.com/reel/Cn0MordNOio/?utm_source=ig_web_copy_link&igsh=MzRlODBiNWFlZA==	21/03/2025
Ação da Cidadania	24/01/2023	Post divulgando a doação de alimentos e campanha para arrecadar recursos através de matéria jornalística	https://www.instagram.com/reel/Cny6NajJDM-/?utm_source=ig_web_copy_link&igsh=MzRlODBiNWFlZA==	21/03/2025
Ação da Cidadania	23/01/2023	Post divulgando a doação de alimentos e campanha para arrecadar recursos	https://www.instagram.com/p/Cnxnw-pP9qK/?utm_source=ig_web_copy_link&igsh=MzRlODBiNWFlZA==	21/03/2025
Ação da Cidadania	23/01/2023	Post divulgando a doação de alimentos e campanha para arrecadar recursos	https://www.instagram.com/p/Cnw4oF7NV2h/?utm_source=ig_web_copy_link&igsh=MzRlODBiNWFlZA==	21/03/2025
Ação da Cidadania	22/01/2023	Post divulgando a doação de alimentos e campanha para arrecadar recursos	https://www.instagram.com/reel/CnuIFoZsUC4/?utm_source=ig_web_copy_link&igsh=MzRlODBiNWFlZA==	21/03/2025
Ação da Cidadania	21/01/2023	Post divulgando a campanha para arrecadar recursos	https://www.instagram.com/p/CnsSgzIpSzz/?utm_source=ig_web_copy_link&igsh=MzRlODBiNWFlZA==	21/03/2025
Ação da Cidadania	2023	Divulgação da ação desenvolvida no território	https://uploads.strikinglycdn.com/files/6d349699-75cc-48b8-acbb-740419d2f0da/	21/03/2025

		Yanomami	relatorio2023.pdf	
Agência Adventista de Desenvolvimento e Recursos Assistenciais (ADRA)	14/03/2023	Divulgação da ação desenvolvida no território Yanomami	https://www.instagram.com/p/CpyIq4bO5rK/?utm_source=ig_web_copy_link&igsh=MzRlODBiNWFlZA==	21/03/2025
ADRA	13/02/2023	Post sobre entrega de filtros de água para a Casa de assistência indígena.	https://www.instagram.com/p/ConhPvYvysD/?utm_source=ig_web_copy_link&igsh=MzRlODBiNWFlZA==	21/03/2025
ADRA	07/02/2023	Divulgação da campanha de doação de filtros de água e arrecadação de recursos financeiros	https://www.instagram.com/p/CoXhck8O0lh/?utm_source=ig_web_copy_link&igsh=MzRlODBiNWFlZA==	21/03/2025
ADRA	03/02/2023	Divulgação das ações desenvolvidas no território Yanomami e na disponibilização de duas nutricionistas para atuar na emergência	https://www.instagram.com/p/CoNtKnBSB09/?utm_source=ig_web_copy_link&igsh=MzRlODBiNWFlZA==	21/03/2025
ADRA	02/02/2023	Divulgação da campanha de doação de filtros de água e arrecadação de recursos financeiros	https://www.instagram.com/p/CoLJA1aukWV/?utm_source=ig_web_copy_link&igsh=MzRlODBiNWFlZA==	21/03/2025
ADRA	24/01/2023	Divulgação da campanha de doação de filtros de água e alimentos e arrecadação de recursos financeiros	https://www.instagram.com/p/Cnz92g1vxdU/?utm_source=ig_web_copy_link&igsh=MzRlODBiNWFlZA==	21/03/2025
ADRA	12/09/2024	Edital para convocação de voluntário para atuar junto ao Fundo das Nações Unidas para Infância (Unicef) no projeto WASH	https://files.adventistas.org/v2.adra.org.br/2024/09/18122459/RR-015-PROCESSO-SELETIVO-UNICEF-ADRA.pdf	21/03/2025
ADRA	15/08/2024	Edital para convocação de voluntário nutricionista para atuar na emergência Yanomami	https://files.adventistas.org/v2.adra.org.br/2024/08/15154637/RR-011-PROCESSO-SELETIVO-ADRA-UNICEF-2024.pdf	21/03/2025
ADRA	01/02/2024	Edital para convocação de voluntário nutricionista para atuar na emergência Yanomami	https://files.adventistas.org/v2.adra.org.br/2024/02/01175943/RR-001-PROCESSO-SELETIVO-ADRA-UNICEF-2024.pdf	21/03/2025
Água Camelo	28/12/2023	Post sobre as ações desenvolvidas no ano de 2023 com menção a ação no território Yanomami e rede de parceiros	https://www.instagram.com/p/C1ZqY-oJlR5/?utm_source=ig_web_copy_link&igsh=MzRlODBiNWFlZA==	21/03/2025
Água Camelo	07/12/2023	Post comemorativo de prêmio recebido pela ação desenvolvida no Território Yanomami e agradecimento aos parceiros	https://www.instagram.com/p/C0j_12TJRyL/?utm_source=ig_web_copy_link&igsh=MzRlODBiNWFlZA==	21/03/2025
Água Camelo	13/02/2023	Post divulgando a doação dos kits de água através de matéria jornalística	https://www.instagram.com/p/CongSXBJj3n/?utm_source=ig_web_copy_link&igsh=MzRlODBiNWFlZA==	21/03/2025
Água Camelo	06/02/2023	Apresentação dos materiais doados para presidente da Fundação Nacional dos Povos Indígenas (Funai)	https://www.instagram.com/reel/CoVL-sjJjSH/?utm_source=ig_web_copy_link&igsh=MzRlODBiNWFlZA==	21/03/2025
Água Camelo	05/02/2023	Post divulgando a doação dos kits de água através de matéria jornalística	https://www.instagram.com/reel/CoS0OYQpJln/?utm_source=ig_web_copy_link&igsh=MzRlODBiNWFlZA==	21/03/2025
Água Camelo	03/02/2023	Post divulgando a doação dos kits de água através de matéria jornalística	https://www.instagram.com/p/CoN2gALJ9J-/?utm_source=ig_web_copy_link&igsh=MzRlODBiNWFlZA==	21/03/2025

Água Camelo	03/02/2023	Capacitação dos profissionais da Funai para uso dos Kits de água doados	https://www.instagram.com/p/CoNOrrNJBuB/?utm_source=ig_web_copy_link&igsh=MzRlODBiNWFlZA==	21/03/2025
Água Camelo	02/02/2023	Post divulgando a doação dos kits de água através de matéria jornalística	https://www.instagram.com/p/CoK_EV1JsK6/?utm_source=ig_web_copy_link&igsh=MzRlODBiNWFlZA==	21/03/2025
Água Camelo	01/02/2023	Post divulgando a doação dos kits de água através de matéria jornalística	https://www.instagram.com/p/CoIvYw5p7c5/?utm_source=ig_web_copy_link&igsh=MzRlODBiNWFlZA==	21/03/2025
Água Camelo	---	Página oficial sobre o projeto desenvolvido no ano de 2023 no território Yanomami	https://aguacamelo.com.br/yanomami/	21/03/2025
AMBEV	26/02/2023	Divulgação da doação de kits de água para os Yanomami	https://www.instagram.com/p/CpIGIZjPcFs/?utm_source=ig_web_copy_link&igsh=MzRlODBiNWFlZA==	21/03/2025
AMBEV	2024	Divulgação da doação de kits de água para os Yanomami	https://api.mziq.com/mzfilemanager/v2/d/c8182463-4b7e-408c-9d0f-42797662435e/ef00e5eb-ba41-50b4-e4ff-9d8a01ef0a07?origin=1	21/03/2025
Assaí atacadista	31/12/2023	Divulgação da doação de alimentos para os Yanomami	https://api.mziq.com/mzfilemanager/v2/d/ec14f0ab-c5d4-4b12-a413-b6cc7475ed98/e80db46d-f6cf-6c46-808c-93598dcb5e1a?origin=1	21/03/2025
Cáritas Brasileira	14/02/2023	Campanha para arrecadar fundos financeiros para aquisição de alimentos e outros insumos	https://www.instagram.com/p/CopB3PFt0Ie/?utm_source=ig_web_copy_link&igsh=MzRlODBiNWFlZA==	21/03/2025
Conselho indígena de Roraima (CIR)	03/08/2023	Divulgação da doação de alimentos para os Yanomami	https://www.instagram.com/p/CvfQA_fupjz/?utm_source=ig_web_copy_link&igsh=MzRlODBiNWFlZA==	21/03/2025
CIR	31/07/2023	Divulgação da doação de alimentos para os Yanomami	https://www.instagram.com/p/CvXSrjpuX8Q/?utm_source=ig_web_copy_link&igsh=MzRlODBiNWFlZA==	21/03/2025
CIR	28/07/2023	Divulgação da doação de alimentos para os Yanomami	https://www.instagram.com/reel/CvP32qvx6rG/?utm_source=ig_web_copy_link&igsh=MzRlODBiNWFlZA==	21/03/2025
CIR	28/07/2023	Divulgação da doação de alimentos para os Yanomami	https://www.instagram.com/reel/CvP3O3NAeFX/?utm_source=ig_web_copy_link&igsh=MzRlODBiNWFlZA==	21/03/2025
CIR	28/07/2023	Divulgação da doação de alimentos para os Yanomami	https://www.instagram.com/p/CvP2IYku4qT/?utm_source=ig_web_copy_link&igsh=MzRlODBiNWFlZA==	21/03/2025
CIR	30/06/2023	Divulgação da doação de alimentos para os Yanomami	https://www.instagram.com/reel/CuIgvM-MtIR/?utm_source=ig_web_copy_link&igsh=MzRlODBiNWFlZA==	21/03/2025
CIR	30/06/2023	Divulgação da doação de alimentos para os Yanomami	https://www.instagram.com/p/CuId5ARuEs0/?utm_source=ig_web_copy_link&igsh=MzRlODBiNWFlZA==	21/03/2025
CIR	12/04/2023	Divulgação da doação de alimentos para os Yanomami	https://www.instagram.com/p/Cq9JAV6JU6O/?utm_source=ig_web_copy_link&igsh=MzRlODBiNWFlZA==	21/03/2025
CIR	12/04/2023	Divulgação da doação de alimentos para os Yanomami	https://www.instagram.com/reel/Cq9AqbPs4iq/?utm_source=ig_web_copy_link&igsh=MzRlODBiNWFlZA==	21/03/2025
CIR	20/02/2023	Divulgação da doação de alimentos para os Yanomami	https://www.instagram.com/p/Co5e8z8pFW-/?utm_source=ig_web_copy_link&igsh=MzRlODBiNWFlZA==	21/03/2025
CIR	11/02/2023	Divulgação da campanha para arrecadar fundos financeiros para aquisição de alimentos e outros insumos	https://www.instagram.com/p/Cohf76FuNJn/?utm_source=ig_web_copy_link&igsh=MzRlODBiNWFlZA==	21/03/2025

CIR	20/02/2023	Divulgação da campanha para arrecadar fundos financeiros para aquisição de alimentos e outros insumos	https://www.cir.org.br/post/sos-yanomami-campanha-ja-distribuiu-cinco-toneladas-de-alimentacao-nas-regioes-de-ajanari-surucucu-xexena-e-auwaris	21/03/2025
Coordenação das Organizações Indígenas da Amazônia Brasileira (COIAB)	13/04/2023	Divulgação da doação de alimentos para os Yanomami	https://www.instagram.com/p/Cq-TSBXNwrB/?utm_source=ig_web_copy_link&igsh=MzRlODBiNWFlZA==	21/03/2025
COIAB	17/02/2023	Divulgação da doação de alimentos para os Yanomami	https://www.instagram.com/p/Cox3DRhpeHI/?utm_source=ig_web_copy_link&igsh=MzRlODBiNWFlZA==	21/03/2025
COIAB	26/01/2023	Campanha para arrecadar recursos financeiros a serem revertidos em alimento	https://www.instagram.com/p/Cn46rWqNHY7/?utm_source=ig_web_copy_link&igsh=MzRlODBiNWFlZA==	22/03/2025
Cozinha solidária do Movimento dos Trabalhadores Sem-Teto (MTST)	25/01/2023	Campanha para arrecadar recursos financeiros e alimentos	https://www.instagram.com/p/Cn1vItmO8eK/?utm_source=ig_web_copy_link&igsh=MzRlODBiNWFlZA==	22/03/2025
Central Única das Favelas (CUFA)	12/02/2023	Post divulgando a doação de alimentos através de matéria jornalística	https://www.instagram.com/p/ColSe4UpMzP/?utm_source=ig_web_copy_link&igsh=MzRlODBiNWFlZA==	22/03/2025
CUFA	11/02/2023	Post divulgando a doação de alimentos através de matéria jornalística	https://www.instagram.com/reel/CohjxnapOoh/?utm_source=ig_web_copy_link&igsh=MzRlODBiNWFlZA==	22/03/2025
CUFA	09/02/2023	Divulgação de doação de alimentos para os Yanomami	https://www.instagram.com/p/CocdSOFrPuc/?utm_source=ig_web_copy_link&igsh=MzRlODBiNWFlZA==	22/03/2025
CUFA	09/02/2023	Divulgação de doação de alimentos para os Yanomami	https://www.instagram.com/reel/CocTyiQPWC1/?utm_source=ig_web_copy_link	22/03/2025
CUFA	08/02/2023	Divulgação de doação de alimentos para os Yanomami, em parceria com o Palmeiras	https://www.instagram.com/reel/CoaSXU-p62e/?utm_source=ig_web_copy_link&igsh=MzRlODBiNWFlZA==	22/03/2025
CUFA	07/02/2023	Divulgação de doação de alimentos para os Yanomami	https://www.instagram.com/p/CoYgu5OpSS7/?utm_source=ig_web_copy_link&igsh=MzRlODBiNWFlZA==	22/03/2025
CUFA	07/02/2023	Divulgação de doação de alimentos para os Yanomami	https://www.instagram.com/reel/CoYZTptJa_-/?utm_source=ig_web_copy_link&igsh=MzRlODBiNWFlZA==	22/03/2025
CUFA	05/02/2023	Post divulgando a doação dos kits de água através de matéria jornalística	https://www.instagram.com/reel/CoSJL4LOndz/?utm_source=ig_web_copy_link&igsh=MzRlODBiNWFlZA==	22/03/2025
CUFA	04/02/2023	Divulgação de doação de alimentos pelo Palmeiras, através de matéria jornalística	https://www.instagram.com/p/CoPpBJHuMC9/?utm_source=ig_web_copy_link&igsh=MzRlODBiNWFlZA==	22/03/2025
CUFA	02/02/2023	Divulgação de doação de alimentos para os Yanomami	https://www.instagram.com/reel/CoLXkURJ0Fs/?utm_source=ig_web_copy_link&igsh=MzRlODBiNWFlZA==	22/03/2025
CUFA	02/02/2023	Divulgação de doação de alimentos para os Yanomami	https://www.instagram.com/reel/CoK2aqKpPJe/?utm_source=ig_web_copy_link&igsh=MzRlODBiNWFlZA==	22/03/2025
CUFA	01/02/2023	Divulgação de doação de alimentos para os Yanomami	https://www.instagram.com/reel/CoIMkJQpoVv/?utm_source=ig_web_copy_link&igsh=MzRlODBiNWFlZA==	22/03/2025
CUFA	01/02/2023	Divulgação de doação de alimentos para os Yanomami	https://www.instagram.com/reel/CoH9ewkNgbN/?utm_source=ig_web_copy_link&igsh=MzRlODBiNWFlZA==	22/03/2025

CUFA	01/02/2023	Divulgação de doação de alimentos para os Yanomami	https://www.instagram.com/p/CoHw8MFrhQK/?utm_source=ig_web_copy_link&igsh=MzRlODBiNWFlZA==	22/03/2025
CUFA	31/01/2023	Post divulgando a doação dos kits de água	https://www.instagram.com/p/CoF6ITapn7h/?utm_source=ig_web_copy_link	22/03/2025
CUFA	31/01/2023	Post divulgando a doação dos kits de água	https://www.instagram.com/p/CoFYHBKLnY6/?utm_source=ig_web_copy_link&igsh=MzRlODBiNWFlZA==	22/03/2025
CUFA	30/01/2023	Post divulgando a doação de alimentos através de matéria jornalística	https://www.instagram.com/reel/CoD5Uc2JSDv/?utm_source=ig_web_copy_link&igsh=MzRlODBiNWFlZA==	22/03/2025
CUFA	30/01/2023	Post divulgando a doação dos kits de água através de matéria jornalística	https://www.instagram.com/p/CoDw2CeNrku/?utm_source=ig_web_copy_link&igsh=MzRlODBiNWFlZA==	22/03/2025
CUFA	30/01/2023	Post divulgando a doação dos kits de água através de matéria jornalística	https://www.instagram.com/p/CoDCghSpda4/?utm_source=ig_web_copy_link&igsh=MzRlODBiNWFlZA==	22/03/2025
CUFA	29/01/2023	Divulgação de doação de alimentos para os Yanomami	https://www.instagram.com/reel/CoAng65JOmE/?utm_source=ig_web_copy_link&igsh=MzRlODBiNWFlZA==	22/03/2025
CUFA	28/01/2023	Divulgação de doação de alimentos e kits para filtrar água para os Yanomami	https://www.instagram.com/p/Cn-LMjcJlWj/?utm_source=ig_web_copy_link&igsh=MzRlODBiNWFlZA==	22/03/2025
CUFA	28/01/2023	Divulgação de doação de alimentos para os Yanomami	https://www.instagram.com/reel/Cn96MBzJx32/?utm_source=ig_web_copy_link&igsh=MzRlODBiNWFlZA==	22/03/2025
CUFA	28/01/2023	Divulgação de doação de alimentos para os Yanomami	https://www.instagram.com/p/Cn9xIXmJDn7/?utm_source=ig_web_copy_link&igsh=MzRlODBiNWFlZA==	22/03/2025
CUFA	28/01/2023	Divulgação de doação de alimentos para os Yanomami	https://www.instagram.com/p/Cn9uyuhJuHN/?utm_source=ig_web_copy_link&igsh=MzRlODBiNWFlZA==	22/03/2025
CUFA	28/01/2023	Divulgação de doação de alimentos para os Yanomami, através de matéria jornalística	https://www.instagram.com/p/Cn9kLl7u1Ev/?utm_source=ig_web_copy_link&igsh=MzRlODBiNWFlZA==	22/03/2025
CUFA	28/01/2023	Post divulgando a doação dos kits de água	https://www.instagram.com/reel/Cn9K-Hos7Kg/?utm_source=ig_web_copy_link&igsh=MzRlODBiNWFlZA==	22/03/2025
CUFA	27/01/2023	Divulgação de doação de alimentos para os Yanomami	https://www.instagram.com/p/Cn8H6eKpTyt/?utm_source=ig_web_copy_link&igsh=MzRlODBiNWFlZA==	22/03/2025
CUFA	27/01/2023	Divulgação de doação de alimentos para os Yanomami	https://www.instagram.com/p/Cn8C1MNpMGa/?utm_source=ig_web_copy_link&igsh=MzRlODBiNWFlZA==	22/03/2025
CUFA	27/01/2023	Divulgação de doação de alimentos para os Yanomami	https://www.instagram.com/reel/Cn7_SsPJzKV/?utm_source=ig_web_copy_link&igsh=MzRlODBiNWFlZA==	22/03/2025
CUFA	27/01/2023	Divulgação de doação de alimentos para os Yanomami	https://www.instagram.com/p/Cn79WvmpkBR/?utm_source=ig_web_copy_link&igsh=MzRlODBiNWFlZA==	22/03/2025
CUFA	27/01/2023	Divulgação de doação de alimentos para os Yanomami	https://www.instagram.com/reel/Cn75xHcJ97m/?utm_source=ig_web_copy_link&igsh=MzRlODBiNWFlZA==	22/03/2025
CUFA	27/01/2023	Divulgação de doação de alimentos para os Yanomami	https://www.instagram.com/p/Cn7LADpJItX/?utm_source=ig_web_copy_link&igsh=MzRlODBiNWFlZA==	22/03/2025
CUFA	27/01/2023	Divulgação de doação de alimentos para os Yanomami, através de matéria jornalística	https://www.instagram.com/reel/Cn7C3UeN_9Y/?utm_source=ig_web_copy_link&igsh=MzRlODBiNWFlZA==	22/03/2025

CUFA	27/01/2023	Divulgação de doação de alimentos para os Yanomami, através de matéria jornalística	https://www.instagram.com/p/Cn65CbHOXky/?utm_source=ig_web_copy_link&igsh=MzRlODBiNWFlZA==	22/03/2025
CUFA	27/01/2023	Divulgação de doação de alimentos para os Yanomami, através de matéria jornalística	https://www.instagram.com/reel/Cn5ifkPJCSj/?utm_source=ig_web_copy_link&igsh=MzRlODBiNWFlZA==	22/03/2025
CUFA	26/01/2023	Divulgação de doação de alimentos para os Yanomami	https://www.instagram.com/reel/Cn5LxBdpH9j/?utm_source=ig_web_copy_link&igsh=MzRlODBiNWFlZA==	22/03/2025
CUFA	26/01/2023	Divulgação de doação de alimentos para os Yanomami, através de matéria jornalística	https://www.instagram.com/reel/Cn43N8xJKUr/?utm_source=ig_web_copy_link&igsh=MzRlODBiNWFlZA==	22/03/2025
CUFA	26/01/2023	Divulgação de doação de alimentos para os Yanomami, através de matéria jornalística	https://www.instagram.com/p/Cn40OB-ub_u/?utm_source=ig_web_copy_link&igsh=MzRlODBiNWFlZA==	22/03/2025
CUFA	25/01/2023	Divulgação de doação de alimentos para os Yanomami, através de matéria jornalística	https://www.instagram.com/tv/Cn27yT7JxSz/?utm_source=ig_web_copy_link&igsh=MzRlODBiNWFlZA==	22/03/2025
CUFA	25/01/2023	Divulgação de doação de alimentos para os Yanomami	https://www.instagram.com/p/Cn2xJo-pSu9/?utm_source=ig_web_copy_link&igsh=MzRlODBiNWFlZA==	22/03/2025
CUFA	25/01/2023	Divulgação de doação de alimentos para os Yanomami, através de matéria jornalística	https://www.instagram.com/reel/Cn2TC6qpUcW/?utm_source=ig_web_copy_link&igsh=MzRlODBiNWFlZA==	22/03/2025
CUFA	24/01/2023	Divulgação de doação de alimentos para os Yanomami, através de matéria jornalística	https://www.instagram.com/reel/Cn0f_5DDHMx/?utm_source=ig_web_copy_link&igsh=MzRlODBiNWFlZA==	22/03/2025
CUFA	24/01/2023	Divulgação de doação de alimentos para os Yanomami, através de matéria jornalística	https://www.instagram.com/reel/Cn0OgDmJEI2/?utm_source=ig_web_copy_link	22/03/2025
CUFA	24/01/2023	Divulgação de doação de alimentos para os Yanomami, através de matéria jornalística	https://www.instagram.com/reel/Cn0EYZfpxJS/?utm_source=ig_web_copy_link&igsh=MzRlODBiNWFlZA==	22/03/2025
CUFA	24/01/2023	Divulgação de doação de alimentos para os Yanomami, através de matéria jornalística	https://www.instagram.com/reel/Cn0B71iJ0AT/?utm_source=ig_web_copy_link&igsh=MzRlODBiNWFlZA==	22/03/2025
CUFA	24/01/2023	Divulgação de doação de alimentos para os Yanomami, através de matéria jornalística	https://www.instagram.com/reel/Cnz4kqaJNLa/?utm_source=ig_web_copy_link&igsh=MzRlODBiNWFlZA==	22/03/2025
CUFA	24/01/2023	Divulgação de doação de alimentos para os Yanomami, através de matéria jornalística	https://www.instagram.com/reel/Cnz4C8SJKri/?utm_source=ig_web_copy_link&igsh=MzRlODBiNWFlZA==	22/03/2025
CUFA	24/01/2023	Divulgação de doação de alimentos para os Yanomami, através de matéria jornalística	https://www.instagram.com/p/CnzfDWer1ox/?utm_source=ig_web_copy_link&igsh=MzRlODBiNWFlZA==	22/03/2025
CUFA	24/01/2023	Divulgação de doação de alimentos para os Yanomami, através de matéria jornalística	https://www.instagram.com/reel/Cnza83Us3Dr/?utm_source=ig_web_copy_link&igsh=MzRlODBiNWFlZA==	22/03/2025
CUFA	24/01/2023	Divulgação de doação de alimentos para os Yanomami	https://www.instagram.com/p/CnzO2_HrL64/?utm_source=ig_web_copy_link&igsh=MzRlODBiNWFlZA==	22/03/2025

CUFA	24/01/2023	Divulgação de doação de alimentos para os Yanomami	https://www.instagram.com/p/CnzJaBYLKML/?utm_source=ig_web_copy_link&igsh=MzRlODBiNWFlZA==	22/03/2025
CUFA	24/01/2023	Divulgação de doação de alimentos para os Yanomami, através de matéria jornalística	https://www.instagram.com/reel/CnzFgwGvqC-/?utm_source=ig_web_copy_link&igsh=MzRlODBiNWFlZA==	22/03/2025
CUFA	24/01/2023	Divulgação de doação de alimentos para os Yanomami, através de matéria jornalística	https://www.instagram.com/reel/CnzEz8KtKsC/?utm_source=ig_web_copy_link&igsh=MzRlODBiNWFlZA==	22/03/2025
CUFA	23/01/2023	Divulgação de doação de alimentos para os Yanomami, através de matéria jornalística	https://www.instagram.com/reel/Cnx_nHtMeO0/?utm_source=ig_web_copy_link&igsh=MzRlODBiNWFlZA==	22/03/2025
CUFA	23/01/2023	Divulgação de doação de alimentos para os Yanomami, através de matéria jornalística	https://www.instagram.com/reel/CnxM1AVvNY1/?utm_source=ig_web_copy_link&igsh=MzRlODBiNWFlZA==	22/03/2025
CUFA	22/01/2023	Campanha para arrecadar recursos financeiros a serem revertidos em alimento	https://cufa.org.br/cufa-e-frente-nacional-antirracista-lancam-campanha-para-ajudar-os-yanomami-e-regiao/	22/03/2025
CUFA Roraima	19/04/2023	Divulgação da doação de alimentos para os Yanomami	https://www.instagram.com/p/CrO3c4fudAY/?utm_source=ig_web_copy_link&igsh=MzRlODBiNWFlZA==	22/03/2025
CUFA Roraima	12/02/2023	Post divulgando a doação de alimentos através de matéria jornalística	https://www.instagram.com/p/ColSe4UpMzP/?utm_source=ig_web_copy_link&igsh=MzRlODBiNWFlZA==	22/03/2025
CUFA Roraima	11/02/2023	Post divulgando a doação de alimentos através de matéria jornalística	https://www.instagram.com/reel/CoiHh1-NMRY/?utm_source=ig_web_copy_link&igsh=MzRlODBiNWFlZA==	22/03/2025
CUFA Roraima	09/02/2023	Divulgação da doação de alimentos para os Yanomami	https://www.instagram.com/p/Cocjrc6O6Bn/?utm_source=ig_web_copy_link&igsh=MzRlODBiNWFlZA==	22/03/2025
CUFA Roraima	09/02/2023	Divulgação da doação de alimentos para os Yanomami	https://www.instagram.com/p/CocZhlQOrI_/?utm_source=ig_web_copy_link&igsh=MzRlODBiNWFlZA==	22/03/2025
CUFA Roraima	07/02/2023	Divulgação da doação de alimentos para os Yanomami	https://www.instagram.com/p/CoYgu5OpSS7/?utm_source=ig_web_copy_link&igsh=MzRlODBiNWFlZA==	22/03/2025
CUFA Roraima	07/02/2023	Divulgação da doação de alimentos para os Yanomami	https://www.instagram.com/reel/CoYZTptJa_-/?utm_source=ig_web_copy_link&igsh=MzRlODBiNWFlZA==	22/03/2025
CUFA Roraima	02/02/2023	Divulgação da doação de alimentos para os Yanomami	https://www.instagram.com/reel/CoLXkURJ0Fs/?utm_source=ig_web_copy_link&igsh=MzRlODBiNWFlZA==	22/03/2025
CUFA Roraima	01/02/2023	Divulgação da doação de alimentos para os Yanomami	https://www.instagram.com/reel/CoH9ewkNgbN/?utm_source=ig_web_copy_link&igsh=MzRlODBiNWFlZA==	22/03/2025
CUFA Roraima	01/02/2023	Divulgação da doação de alimentos para os Yanomami	https://www.instagram.com/p/CoHw8MFrhQK/?utm_source=ig_web_copy_link&igsh=MzRlODBiNWFlZA==	22/03/2025
CUFA Roraima	31/01/2023	Divulgação da doação de alimentos e da campanha para arrecadar alimentos para os Yanomami	https://www.instagram.com/p/CoF6ITapn7h/?utm_source=ig_web_copy_link&igsh=MzRlODBiNWFlZA==	22/03/2025
CUFA Roraima	31/01/2023	Divulgação da doação de alimentos e da campanha para arrecadar alimentos para os Yanomami	https://www.instagram.com/p/CoFYHBKLnY6/?utm_source=ig_web_copy_link&igsh=MzRlODBiNWFlZA==	22/03/2025
CUFA Roraima	30/01/2023	Divulgação da doação de	https://www.instagram.com/p/CoDCghSpda4/?	22/03/2025

		kits para tratar água	utm_source=ig_web_copy_link&igsh=MzRlODBiNWFlZA==	
CUFA Roraima	28/01/2023	Divulgação de parceria com a Funai e atores comerciais	https://www.instagram.com/p/Cn-LMjcJlWj/?utm_source=ig_web_copy_link&igsh=MzRlODBiNWFlZA==	22/03/2025
CUFA Roraima	28/01/2023	Divulgação da doação de alimentos e da campanha para arrecadar recursos financeiros para os Yanomami	https://www.instagram.com/reel/Cn99RnZury_/?utm_source=ig_web_copy_link&igsh=MzRlODBiNWFlZA==	22/03/2025
CUFA Roraima	28/01/2023	Divulgação da doação de alimentos e da campanha para arrecadar recursos financeiros para os Yanomami	https://www.instagram.com/reel/Cn96MBzJx32/?utm_source=ig_web_copy_link&igsh=MzRlODBiNWFlZA==	22/03/2025
CUFA Roraima	28/01/2023	Divulgação da doação de alimentos	https://www.instagram.com/p/Cn9w75NOwF4/?utm_source=ig_web_copy_link&igsh=MzRlODBiNWFlZA==	22/03/2025
CUFA Roraima	28/01/2023	Divulgação da doação de alimentos	https://www.instagram.com/p/Cn9uyuhJuHN/?utm_source=ig_web_copy_link&igsh=MzRlODBiNWFlZA==	22/03/2025
CUFA Roraima	28/01/2023	Divulgação da doação de alimentos através de matéria jornalística	https://www.instagram.com/p/Cn9kLEwOwYK/?utm_source=ig_web_copy_link&igsh=MzRlODBiNWFlZA==	22/03/2025
CUFA Roraima	28/01/2023	Divulgação da doação de alimentos	https://www.instagram.com/reel/Cn9bc1CNBY-/?utm_source=ig_web_copy_link&igsh=MzRlODBiNWFlZA==	22/03/2025
CUFA Roraima	28/01/2023	Divulgação da doação de alimentos	https://www.instagram.com/p/Cn9a6bULh_K/?utm_source=ig_web_copy_link&igsh=MzRlODBiNWFlZA==	22/03/2025
CUFA Roraima	28/01/2023	Divulgação da doação de kits para tratar água	https://www.instagram.com/reel/Cn9K-Hos7Kg/?utm_source=ig_web_copy_link&igsh=MzRlODBiNWFlZA==	22/03/2025
CUFA Roraima	27/01/2023	Divulgação da doação de alimentos	https://www.instagram.com/p/Cn8H6eKpTyt/?utm_source=ig_web_copy_link&igsh=MzRlODBiNWFlZA==	22/03/2025
CUFA Roraima	27/01/2023	Divulgação da doação de alimentos e da campanha para arrecadar recursos financeiros para os Yanomami	https://www.instagram.com/p/Cn8DDGXunCj/?utm_source=ig_web_copy_link&igsh=MzRlODBiNWFlZA==	22/03/2025
CUFA Roraima	27/01/2023	Divulgação da doação de alimentos	https://www.instagram.com/reel/Cn7_SsPJzKV/?utm_source=ig_web_copy_link&igsh=MzRlODBiNWFlZA==	22/03/2025
CUFA Roraima	27/01/2023	Divulgação da doação de alimentos	https://www.instagram.com/p/Cn79WvmpkBR/?utm_source=ig_web_copy_link&igsh=MzRlODBiNWFlZA==	22/03/2025
CUFA Roraima	27/01/2023	Divulgação da doação de alimentos e filtros para água	https://www.instagram.com/p/Cn77JdhONbv/?utm_source=ig_web_copy_link&igsh=MzRlODBiNWFlZA==	22/03/2025
CUFA Roraima	27/01/2023	Divulgação da doação de alimentos	https://www.instagram.com/reel/Cn75xHcJ97m/?utm_source=ig_web_copy_link&igsh=MzRlODBiNWFlZA==	22/03/2025
CUFA Roraima	27/01/2023	Divulgação da doação de alimentos	https://www.instagram.com/p/Cn7LADpJItX/?utm_source=ig_web_copy_link&igsh=MzRlODBiNWFlZA==	22/03/2025
CUFA Roraima	27/01/2023	Divulgação da doação de alimentos	https://www.instagram.com/reel/Cn7FJXsPaZq/?utm_source=ig_web_copy_link&igsh=MzRlODBiNWFlZA==	22/03/2025
CUFA Roraima	27/01/2023	Divulgação da doação de alimentos através de matéria jornalística	https://www.instagram.com/p/Cn666k0OOvS/?utm_source=ig_web_copy_link&igsh=MzRlODBiNWFlZA==	22/03/2025
CUFA Roraima	26/01/2023	Divulgação da doação de	https://www.instagram.com/reel/	22/03/2025

		alimentos através de matéria jornalística	Cn43N8xJKUr/?utm_source=ig_web_copy_link&igsh=MzRlODBiNWFlZA==	
CUFA Roraima	26/01/2023	Divulgação da doação de alimentos	https://www.instagram.com/p/Cn4jcOWOe3H/?utm_source=ig_web_copy_link&igsh=MzRlODBiNWFlZA==	22/03/2025
CUFA Roraima	26/01/2023	Divulgação da doação de alimentos	https://www.instagram.com/p/Cn4iO48OmTs/?utm_source=ig_web_copy_link&igsh=MzRlODBiNWFlZA==	22/03/2025
CUFA Roraima	26/01/2023	Divulgação da doação de alimentos	https://www.instagram.com/reel/Cn4De_qLujl/?utm_source=ig_web_copy_link&igsh=MzRlODBiNWFlZA==	22/03/2025
CUFA Roraima	25/01/2023	Divulgação da doação de alimentos	https://www.instagram.com/reel/Cn3KDe3plT5/?utm_source=ig_web_copy_link&igsh=MzRlODBiNWFlZA==	22/03/2025
CUFA Roraima	25/01/2023	Divulgação da doação de alimentos	https://www.instagram.com/reel/Cn3AkelsseI/?utm_source=ig_web_copy_link&igsh=MzRlODBiNWFlZA==	22/03/2025
CUFA Roraima	25/01/2023	Divulgação da doação de alimentos	https://www.instagram.com/p/Cn2_973ub_O/?utm_source=ig_web_copy_link&igsh=MzRlODBiNWFlZA==	22/03/2025
CUFA Roraima	25/01/2023	Divulgação da doação de alimentos através de matéria jornalística	https://www.instagram.com/reel/Cn2TC6qpUcW/?utm_source=ig_web_copy_link&igsh=MzRlODBiNWFlZA==	22/03/2025
CUFA Roraima	24/01/2023	Divulgação da doação de alimentos através de matéria jornalística	https://www.instagram.com/reel/Cn0f_5DDHMx/?utm_source=ig_web_copy_link&igsh=MzRlODBiNWFlZA==	22/03/2025
CUFA Roraima	24/01/2023	Divulgação da doação de alimentos através de matéria jornalística	https://www.instagram.com/reel/Cnz4kqaJNLa/?utm_source=ig_web_copy_link&igsh=MzRlODBiNWFlZA==	22/03/2025
CUFA Roraima	24/01/2023	Divulgação da doação de alimentos através de matéria jornalística	https://www.instagram.com/reel/Cnz4C8SJKri/?utm_source=ig_web_copy_link&igsh=MzRlODBiNWFlZA==	22/03/2025
CUFA Roraima	24/01/2023	Divulgação da doação de alimentos através de matéria jornalística	https://www.instagram.com/reel/CnzZC4YLAW_/?utm_source=ig_web_copy_link&igsh=MzRlODBiNWFlZA==	22/03/2025
CUFA Roraima	24/01/2023	Divulgação da doação de alimentos	https://www.instagram.com/p/CnzO2_HrL64/?utm_source=ig_web_copy_link&igsh=MzRlODBiNWFlZA==	22/03/2025
CUFA Roraima	24/01/2023	Divulgação da doação de alimentos	https://www.instagram.com/p/CnzJ1azueCd/?utm_source=ig_web_copy_link&igsh=MzRlODBiNWFlZA==	22/03/2025
CUFA Roraima	24/01/2023	Divulgação da doação de alimentos através de matéria jornalística	https://www.instagram.com/p/CnzJaBYLKML/?utm_source=ig_web_copy_link&igsh=MzRlODBiNWFlZA==	22/03/2025
CUFA Roraima	24/01/2023	Divulgação da doação de alimentos através de matéria jornalística	https://www.instagram.com/reel/CnzEz8KtKsC/?utm_source=ig_web_copy_link&igsh=MzRlODBiNWFlZA==	22/03/2025
CUFA Roraima	23/01/2023	Divulgação da doação de alimentos através de matéria jornalística	https://www.instagram.com/reel/CnyCs9Irl05/?utm_source=ig_web_copy_link&igsh=MzRlODBiNWFlZA==	22/03/2025
CUFA Roraima	23/01/2023	Campanha para arrecadação de recursos financeiros e alimentos	https://www.instagram.com/p/CnxutqNOx9N/?utm_source=ig_web_copy_link&igsh=MzRlODBiNWFlZA==	22/03/2025
CUFA Roraima	23/01/2023	Divulgação da doação de alimentos através de matéria jornalística	https://www.instagram.com/reel/CnxM1AVvNY1/?utm_source=ig_web_copy_link&igsh=MzRlODBiNWFlZA==	22/03/2025
CUFA Roraima	22/01/2023	Campanha para arrecadação de recursos financeiros e alimentos	https://www.instagram.com/p/CnvENKCu4Q_/?utm_source=ig_web_copy_link&igsh=MzRlODBiNWFlZA==	22/03/2025
Diocese de Roraima	10/02/2023	Campanha para	https://www.instagram.com/p/CofuSDouPsO/?	22/03/2025

		arrecadação de recursos financeiros e alimentos	utm_source=ig_web_copy_link&igsh=MzRlODBiNWFlZA==	
Frente Nacional Antirracista (FNA)	26/08/2024	Divulgação da doação de alimentos	https://www.instagram.com/p/C_J7Kdfp0ny/?utm_source=ig_web_copy_link&igsh=MzRlODBiNWFlZA==	22/03/2025
FNA	11/02/2023	Divulgação da doação de alimentos através de matéria jornalística	https://www.instagram.com/reel/CohjxnapOoh/?utm_source=ig_web_copy_link&igsh=MzRlODBiNWFlZA==	22/03/2025
FNA	29/01/2023	Divulgação da doação de alimentos	https://www.instagram.com/reel/CoAng65JOmE/?utm_source=ig_web_copy_link&igsh=MzRlODBiNWFlZA==	22/03/2025
FNA	26/01/2023	Divulgação da doação de alimentos	https://www.instagram.com/reel/Cn5LxBdpH9j/?utm_source=ig_web_copy_link&igsh=MzRlODBiNWFlZA==	22/03/2025
FNA	26/01/2023	Divulgação da doação de alimentos e da campanha para arrecadar fundos	https://www.instagram.com/reel/Cn4d3ElA6HO/?utm_source=ig_web_copy_link&igsh=MzRlODBiNWFlZA==	22/03/2025
FNA	25/01/2023	Divulgação da doação de alimentos	https://www.instagram.com/p/Cn22ehtPigV/?utm_source=ig_web_copy_link	22/03/2025
FNA	25/01/2023	Divulgação da doação de alimentos	https://www.instagram.com/p/Cn1e66yOIlL/?utm_source=ig_web_copy_link&igsh=MzRlODBiNWFlZA==	22/03/2025
FNA	24/01/2023	Divulgação da doação de alimentos através de matéria jornalística	https://www.instagram.com/reel/Cn0mGLXgNZ6/?utm_source=ig_web_copy_link&igsh=MzRlODBiNWFlZA==	22/03/2025
FNA	24/01/2023	Divulgação da doação de alimentos através de matéria jornalística	https://www.instagram.com/reel/Cn0jiVBgMQI/?utm_source=ig_web_copy_link&igsh=MzRlODBiNWFlZA==	22/03/2025
FNA	24/01/2023	Divulgação da doação de alimentos e arrecadação de fundos	https://www.instagram.com/reel/Cn0TzMIA_fh/?utm_source=ig_web_copy_link&igsh=MzRlODBiNWFlZA==	22/03/2025
FNA	24/01/2023	Divulgação da doação de alimentos e arrecadação de fundos	https://www.instagram.com/p/CnzFTqWOztN/?utm_source=ig_web_copy_link&igsh=MzRlODBiNWFlZA==	22/03/2025
FNA	24/01/2023	Divulgação da doação de alimentos e arrecadação de fundos através de matéria jornalística	https://www.instagram.com/reel/CnzETmPgMxj/?utm_source=ig_web_copy_link&igsh=MzRlODBiNWFlZA==	22/03/2025
FNA	24/01/2023	Divulgação da doação de alimentos e arrecadação de fundos através de matéria jornalística	https://www.instagram.com/reel/CnyVEingOKq/?utm_source=ig_web_copy_link&igsh=MzRlODBiNWFlZA==	22/03/2025
FNA	24/01/2023	Divulgação da doação de alimentos e arrecadação de fundos através de matéria jornalística	https://www.instagram.com/reel/CnyUkLzgIhI/?utm_source=ig_web_copy_link&igsh=MzRlODBiNWFlZA==	22/03/2025
Fundação José Luiz Egydio Setúbal - Instituto Pensi	07/02/2024	Divulgação de doação de fórmulas terapêuticas para o Unicef	https://institutopensi.org.br/uma-pequena-ajuda-humanitaria-aos-yanomamis/	27/03/2025
iFood	2025	Divulgação da doação de recursos para a CUFA	https://institucional.ifood.com.br/wp-content/uploads/2025/02/Ifood_Relatorio_Trimestral-3T24_atualizado.pdf	27/03/2025
iFood	2024	Divulgação da doação de recursos para a CUFA, convertidos em cesta básica para os yanomami	https://institucional.ifood.com.br/wp-content/uploads/2024/01/Ifood_info_transparencia_23_1Trimestre_.pdf	27/03/2025
Instituto A nossa jornada	02/02/2023	Divulgação da doação de alimentos para os Yanomami	https://www.instagram.com/p/CoKJwKlufoM/?utm_source=ig_web_copy_link&igsh=MzRlODBiNWFlZA==	27/03/2025
Instituto A nossa jornada	20/01/2023	Divulgação da doação de alimentos para os	https://www.instagram.com/p/Cnp92q1Pasg/?utm_source=ig_web_copy_link&igsh=MzRlOD	27/03/2025

		Yanomami	BiNWFlZA==	
Instituto Assaí	2024	Divulgação da doação de alimentos para os Yanomami	https://api.mziq.com/mzfilemanager/v2/d/ec14f0ab-c5d4-4b12-a413-b6cc7475ed98/460316e6-5d6e-11d7-5a02-9be4d9bc0bee?origin=1	27/03/2025
Instituto Assaí	26/01/2023	Divulgação da doação de alimentos para os Yanomami	https://www.instagram.com/p/Cn5PSGzJpQ8/?utm_source=ig_web_copy_link	27/03/2025
Instituto C&A	2024	Divulgação da doação de alimentos para os Yanomami, através da Ação da Cidadania	https://drive.google.com/file/d/1fddj1k-AVKMmeNFV3_oCUAttuCLfdnSV/view	27/03/2025
Instituto C&A	26/01/2023	Divulgação da doação de alimentos para os Yanomami, através da Ação da Cidadania	https://www.instagram.com/p/Cn5V-zYPBEe/?utm_source=ig_web_copy_link&igsh=MzRlODBiNWFlZA==	27/03/2025
Palmeiras	07/02/2023	Divulgação da doação de alimentos para os Yanomami, através da CUFA	https://www.instagram.com/reel/CoYMS1rrJ8L/?utm_source=ig_web_copy_link&igsh=MzRlODBiNWFlZA==	27/03/2025
Palmeiras	02/02/2023	Campanha para arrecadar alimentos e recursos financeiros para os Yanomami, através da CUFA	https://www.instagram.com/p/CoLKAYYrobK/?utm_source=ig_web_copy_link	27/03/2025
Palmeiras	07/02/2023	Divulgação da doação de alimentos para os Yanomami, através da CUFA	https://www.palmeiras.com.br/noticias/palmeiras-vai-arrecadar-alimentos-para-o-povo-yanomami-em-jogo-no-allianz-parque/	27/03/2025
Palmeiras	02/02/2023	Divulgação da doação de alimentos para os Yanomami, através da CUFA	https://www.palmeiras.com.br/noticias/por-um-futuro-mais-verde-palmeiras-e-torcedores-doam-alimentos-ao-povo-yanomami/	27/03/2025
Real Cestas	30/01/2023	Divulgação de parceria com a CUFA para distribuir cestas básicas para os Yanomami, através de matéria jornalística	https://www.instagram.com/reel/CoDLPDuJk7E/?utm_source=ig_web_copy_link&igsh=MzRlODBiNWFlZA==	27/03/2025
Real Cestas	27/01/2023	Divulgação de campanha para distribuir cestas básicas para os Yanomami	https://www.instagram.com/p/Cn7XOPIptmh/?utm_source=ig_web_copy_link&igsh=MzRlODBiNWFlZA==	27/03/2025
Serviço Social do Comércio (Sesc)	11/07/2024	Divulgação da doação de alimentos para os Yanomami	https://www.instagram.com/p/C9SyZP3iHuO/?utm_source=ig_web_copy_link&igsh=MzRlODBiNWFlZA==	27/03/2025
Sesc	07/12/2023	Divulgação das ações realizadas ao longo do ano, incluindo as doações de alimentos	https://www.instagram.com/p/C0ji-JQOiHF/?utm_source=ig_web_copy_link&igsh=MzRlODBiNWFlZA==	27/03/2025
Sesc	28/03/2023	Divulgação da doação de alimentos para os Yanomami	https://www.instagram.com/p/CqVfAhGMF2X/?utm_source=ig_web_copy_link&igsh=MzRlODBiNWFlZA==	27/03/2025
Sesc	07/12/2023	Divulgação da doação de alimentos para os Yanomami	https://www.sesc.com.br/noticias/assistencia/sesc-mesa-brasil-entrega-doacoes-ao-povo-indigena-yanomami/	27/03/2025
Sesc	27/03/2023	Divulgação da doação de alimentos para os Yanomami	https://www.sesc.com.br/noticias/assistencia/sesc-faz-doacao-emergencial-aos-yanomami/	27/03/2025
UNICEF	08/04/2024	Divulgação das ações realizadas no território Yanomami	https://www.unicef.org/brazil/apoio-a-resposta-humanitaria-na-terra-indigena-yanomami	27/03/2025
UNICEF	2024	Divulgação das ações realizadas no território Yanomami	https://www.unicef.org/brazil/media/28946/file	27/03/2025
UNICEF	16/03/2023	Divulgação da ação de apoio na recuperação	https://www.instagram.com/p/Cp2UiUIOgig/?utm_source=ig_web_copy_link&igsh=MzRlOD	27/03/2025

		nutricional no território Yanomami	BiNWFlZA==	
UNICEF	05/12/2023	Divulgação da doação de alimento terapêutico	https://www.unicef.org/brazil/innocenti/brazil/comunicados-de-imprensa/unicef-e-fjles-renovam-parceria-em-apoio-a-busca-ativa-vacinal	27/03/2025
UNICEF	03/10/2024	Divulgação de capacitação profissional e criação de cartilhas para identificação e tratamento da desnutrição	https://www.unicef.org/brazil/comunicados-de-imprensa/iniciativa-do-unicef-para-enfrentamento-a-desnutricao-infantil-indigena-visa-impactar-30-mil-criancas-ate-2025	27/03/2025
UNICEF	05/06/2023	Divulgação da ação de apoio na recuperação nutricional no território Yanomami	https://www.unicef.org/brazil/historias/estou-orgulhosa-em-trabalhar-para-esta-grande-familia-yanomami	27/03/2025
UNICEF	24/03/2023	Divulgação de encontro de representantes do Unicef com membros do governo federal	https://www.unicef.org/brazil/comunicados-de-imprensa/representante-do-unicef-se-reune-com-ministra-dos-povos-indigenas#:~:text=Bras%C3%ADlia%2C%2024%20de%20mar%C3%A7o%20de%202023%20%E2%80%93%20A,foco%20nos%20direitos%20de%20crian%C3%A7as%20e%20adolescentes%20ind%C3%ADgenas.	27/03/2025
UNICEF	09/03/2023	Divulgação da ação de apoio na recuperação nutricional no território Yanomami	https://www.unicef.org/brazil/historias/minha-missao-e-cuidar-sinto-os-yanomamis-como-se-fossem-o-meu-proprio-povo	27/03/2025
UNICEF	31/01/2023	Divulgação da ação de apoio na recuperação nutricional no território Yanomami	https://www.unicef.org/brazil/comunicados-de-imprensa/unicef-contribui-para-resposta-%C3%A0-crise-humanit%C3%A1ria-no-territ%C3%B3rio-yanomami	27/03/2025

ADRA: Adventista de Desenvolvimento e Recursos Assistenciais; CIR: Conselho indígena de Roraima; COIAB: Coordenação das Organizações Indígenas da Amazônia Brasileira; CUFA: Central Única das Favelas; FNA: Frente Nacional Antirracista; Funai - Fundação Nacional dos Povos Indígenas; Sesc: Serviço Social do Comércio.

Fonte: elaborado pelos autores.
